# CNS Resident Innate Immune Cells: Guardians of CNS Homeostasis

**DOI:** 10.3390/ijms25094865

**Published:** 2024-04-29

**Authors:** Luca Muzio, Jessica Perego

**Affiliations:** Neuroimmunology Lab, IRCCS San Raffaele Scientific Institute, Institute of Experimental Neurology, 20133 Milan, Italy; perego.jessica@hsr.it

**Keywords:** MS, innate immune cells, brain tissue macrophages, meningeal immunity

## Abstract

Although the CNS has been considered for a long time an immune-privileged organ, it is now well known that both the parenchyma and non-parenchymal tissue (meninges, perivascular space, and choroid plexus) are richly populated in resident immune cells. The advent of more powerful tools for multiplex immunophenotyping, such as single-cell RNA sequencing technique and upscale multiparametric flow and mass spectrometry, helped in discriminating between resident and infiltrating cells and, above all, the different spectrum of phenotypes distinguishing border-associated macrophages. Here, we focus our attention on resident innate immune players and their primary role in both CNS homeostasis and pathological neuroinflammation and neurodegeneration, two key interconnected aspects of the immunopathology of multiple sclerosis.

## 1. Introduction

Multiple sclerosis (MS) is the most prevalent non-resolving chronic inflammatory disease of the central nervous system (CNS), affecting more than 2 million people worldwide [1] with hallmarks of demyelination and axonal damage. MS affects mainly young people between 20 and 40 years old at onset and is characterized by a heterogeneity of symptoms, disease course, and outcomes [2]. Considering the age at disease onset and the target organ, MS emerges as a major cause of disability in young adults in Western countries, posing a significant health and socioeconomic burden [3].

MS has a complex etiology and typically arises in individuals with a genetically predisposed background, influenced by a myriad of environmental factors, such as Epstein-Barr virus infection [4], lifestyle-related factors (physical activity, diet, smoking, etc.), gut microbiota dysbiosis [5], sun exposure, and vitamin D levels [6], as well as hormonal factors linked to puberty and pregnancy [7].

MS is conventionally classified into the relapsing-remitting (RRMS) and progressive (PMS) forms [8]. RRMS is characterized by inflammatory flares followed by a recovery period. It is generally conceived that tissue damage in MS begins early and accumulates as the disease progresses, resulting from a complex interplay among immune cells, glia (myelinating oligodendrocytes, their precursors, microglia, and astrocytes), and neurons [9]. In the last 10 years, more than 15 medications have been approved for modifying the course of MS. Most of them are approved for RRMS but are not effective in the case of PMS. These disease-modifying therapies (DMTs), including immunomodulatory drugs (e.g., interferon-β, dimethyl fumarate), drugs favoring immune reconstitution (e.g., cladribine, ocrelizumab), and immune blockers (e.g., Fingolimod, Natalizumab) can reduce the development of new white-matter lesions, clinical relapses, and thus disability progression in patients [10]. Unfortunately, there is still no efficacious treatment for PMS, and active research is still required to address this unmet clinical need [11].

As with many other CNS pathologies, MS is the result of two main pathological processes: neuroinflammation and neurodegeneration [12]. The prevalent dogma suggests that the main player in MS pathobiology is the inflammatory arm that contributes to the disease with auto-reactive T and B lymphocytes. T cells are activated in the periphery, where they escape tolerance mechanisms. They then undergo clonal expansion and subsequently enter the CNS by crossing the blood-brain barrier (BBB). These T cells can acquire the phenotype of T helper (h)-1, Th17, and Th22, and such differentiation is associated with specific cytokines/chemokines profiles [13]. In the CNS, these T cells receive further stimulation and activate a classical pro-inflammatory response, featuring the release of cytokines such as TNFα, IFNγ, interleukin (IL)-2, IL-12, and IL-17 [14], which in turn drive the activation of CNS resident cells, including microglia and astrocytes.

In this review, we emphasize the significance and intricacy of the landscape of innate immune cells in both neuroinflammation and neurodegeneration. Both pathological mechanisms are also evident in other CNS diseases such as Alzheimer’s, Parkinson’s, or brain ischemia. Although the focus of this review is on MS, some of these neuroinflammatory and neurodegenerative processes can be considered common to a growing number of neurodegenerative disorders.

## 2. MS: Pathophysiology and Tools of Investigation

### 2.1. MS Pathophysiology

MS is a chronic immune-mediated disease of the CNS, characterized by two main components mutually influencing each other: neuroinflammation and neurodegeneration [12]. The outcoming manifestations are motor, sensory, and cognitive dysfunctions, which make MS one of the leading causes of neurological disability in young adults. MS represents an impelling health urgency, with over 2.8 million diagnosed people worldwide and a prevalence that is foreseen to increase over time. Disease relapses followed by a remission phase and the formation of new lesions in the CNS are the classical characteristics of RRMS [15]; namely, the disease form is present in about 85% of patients at diagnosis. In many patients, after 15–20 years, neurodegeneration and chronic inflammation lead to a pattern of steady deterioration referred to as secondary progressive MS (SPMS) [16]. Less than 10% of patients develop a primary progressive form since the beginning (PPMS) [9]. In recent years, MS classical phenotypic characterization has been revised with a more dynamic and multifactorial view of the pathogenesis, in which neuroinflammation—classically associated with RRMS—and neurodegeneration—associated with PMS—coexist. This vision has been recently illustrated by T. Kuhlmann [17], and it is based on the concept that the clinical course of MS is a continuum, with contributions from concurrent pathophysiology varying across individuals and over time. In line with this hypothesis, the shift from RRMS to SPMS is only an apparent evolution reflecting a partial deviation from localized acute injury to widespread inflammation and neurodegeneration. Another key component is the failure of compensatory mechanisms, such as neuroplasticity and remyelination. Altogether, the course of MS is a spectrum given by simultaneous pathological and regenerative mechanisms [17].

### 2.2. Preclinical Models of MS

In addition to the use of magnetic resonance imaging technology, the human CNS is not accessible for investigations. To overcome this limitation, MS research takes advantage of new human in vitro models, as well as established in vivo preclinical models that preferentially involve rodents.

Although human brain organoids are constantly arising and getting closer to the human brain, they are still scarcely recapitulating the whole complexity of the human CNS [18]. Indeed, this “brain-on-a-dish” approach lacks, in most cases, a proper immune system, a vasculature system, a lymphatic system, and the BBB, which are pivotal components of the CNS—not to mention the choroid plexus, the meninges, and the skull bone marrow [19,20].

Thus, the research community working on MS cannot yet avoid taking advantage of rodent’ models for proper in vivo relevant preclinical research. Several models are available; some of them are inducible, while others are genetic mouse models that can still help dissect MS pathobiology [21].

The inducible models comprise the experimental autoimmune encephalomyelitis (EAE) [22] and the cuprizone-induced model for demyelination [23]. MS is an exclusively human disease that does not occur spontaneously in non-human organisms [24]. However, the development of the EAE model, as well as of other animal models, substantially increased the comprehension of MS pathobiology and helped to design new therapeutic strategies. EAE was first described 90 years ago [25], and it is induced by active immunization with peptides derived from myelin proteins emulsified in complete Freund’s adjuvant [26] or adoptive transfer of myelin-specific T cells [27,28], together with pertussis toxin increases the permeability of the BBB [29]. Disease symptoms in the C57BL/6 strain are macroscopically evident around day 10 post-immunization and gradually worsen up to the disease peak, which occurs at around day 20 after immunization. Later, symptoms slightly decrease and eventually stabilize to a lower clinical score compared with the disease peak (chronic phase) [30]. Antigen processing, presentation, and cytokine-mediated steps in secondary lymphoid tissue precede the generation of fully encephalitogenic T cells. Once differentiated, autoimmune Th1 and Th17 enter the CNS and initiate local inflammation. The most used encephalitogenic peptides are the MOG^35-55^ for the C57BL/6 background, while the PLP^135-151^ is used to induce a similar but not identical disease in the SJL mouse strain [22]. EAE is a model that well recapitulates multiple MS features, being a complex condition in which the interaction between immunopathological and neuropathological mechanisms leads to inflammation, demyelination, axonal loss, and gliosis [31]. EAE is considered a good proxy for RRMS since it is characterized by an initial inflammatory flare followed by a chronic phase featuring a slight partial remission. Although EAE does not arise spontaneously, it is induced pharmacologically; the disease onset and severity are sensitive to several environmental factors, including gut microbiota dysbiosis [32]. Despite some limitations that cannot allow the use of the EAE paradigm to reproduce human SPMS or PPMS, this animal model is considered a valid tool for preclinical studies on MS as well as for designing therapeutic tools for RRMS.

The cuprizone model is often used as a preclinical proxy of MS, although it is more appropriate to define it as a tool helping the investigation of demyelination/remyelination processes [33]. Cuprizone [bis(cyclohexanone) oxaldihidrazone] is a copper chelating agent that affects oligodendrocytes metabolism [34,35,36,37,38], although the exact mechanism of myelin degeneration remains unclear. Cuprizone is administered orally with the diet (0.2% *w*/*w*) and leads to oligodendroglia cell death, activation of microglia and astrocytes, and subsequent demyelination, recapitulating MS neurodegeneration and local inflammation [39,40]. This model is particularly instrumental in studying processes involved in demyelination/remyelination processes and tissue regeneration phases, given that cuprizone has a transient effect and spontaneous remyelination occurs after the withdrawal of the neurotoxin [20].

Genetic MS preclinical mouse models, such as the EAE-prone 2D2 and T cell Receptor (TCR)^1640^ transgenic mice, are also useful for dissecting molecular pathways involved in MS pathogenesis. These mice express a MOG^35–55^ specific transgenic TCR on the C57BL/6 background but do not develop MOG-specific B cell response or anti-MOG antibodies [41]. The 2D2 mice were originally ascribed to develop optic neuritis, but they may develop EAE with an incidence of around 1% of the colony [42]. However, this percentage rises to 50% if they undergo i.v. injections of pertussis toxin at a 2-day interval and up to 100% if administered with 50 μg of the MOG-specific IgG1 antibody [41].

TCR^1640^ mice express a TCR recognizing MOG^92-106^ on an SJL/j background and spontaneously develop EAE at a rate of around 90%. This TCR was isolated from an encephalitogenic Th1 cell clone and uses Vα8.3 and Vβ4 genes [43]. This animal model recapitulates well the clinical sex differences that are seen in patients since 80% of females develop a spontaneous disease, which is close to RRMS, while in males, the incidence rate is close to 60%, and the disease form resembles PMS. In addition, contrary to the induced EAE model, where the majority of the neuroinflammation is detected in the spinal cord, in this transgenic mouse model, lesions are present both in the spinal cord and the brain, getting this model closer to the patient’s reality [43].

## 3. Innate Immunity of the CNS: Classification and Ontogeny

### 3.1. CNS Innate Immune Cell Classification

The brain parenchyma innate immune system is composed mainly of myeloid cells belonging to the macrophage family, which are called CNS-associated macrophages (CAMs) [44]. CAMs include microglia, located throughout the brain parenchyma, and three types of border-associated macrophages (BAMs) that are located at the interface between the CNS and BBB [45]. BAMs consist of perivascular macrophages in the perivascular space between the endothelial and parenchymal basement membranes, meningeal macrophages that line the meninges and its vasculature, and macrophages within the choroid plexus. Among the CAMs family, microglia are the most abundantly studied cells, mainly because until recently, it was thought that the microglia were quite the only CNS immune component present within the healthy CNS, working as keepers of physiological conditions.

**Figure 1:**
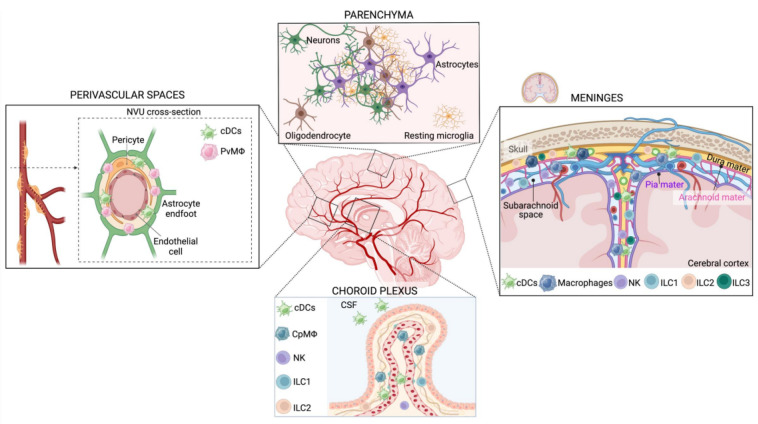
**The CNS innate immune system during homeostasis.** Scheme of the innate immune populations of the brain under physiological conditions, divided by anatomical areas. Brain parenchyma: under physiological conditions, microglia are highly abundant in the CNS. Resting microglia cell morphology features a small cell body with a highly branched ramified morphology. Key phenotypic markers are TMEM119, SALL1, CX3CR1, P2Y12, MerTK, and Iba1. CNS meninges: The meninges are membranes surrounding the brain and the spinal cord: the dura mater, the arachnoid mater, and the pia mater. The arachnoid and pia mater delimit the subarachnoid space and are collectively defined as leptomeninges. BAMs, dendritic cells, and ILCs populate the meninges of the healthy brain. BAMs can be distinguished in subdural (MHC-II low, Egfl7+, and Lyve1+) and dural macrophages (both MHC-II high and low). NKs, ILC1, heterogeneous LTi/LTi-like cells, and NCR+ ILC3s have been found in all three meningeal layers, while ILC2s populate mainly the dura mater but are absent in the leptomeninges. Choroid plexus: The primary function of the choroid plexus is the secretion and modulation of CSF. CpMΦ (ApoE+, Ms4a7+, and Ms4a6c76+) are the largest class of innate immune cells in the choroid plexus and are predominantly associated with blood vessels. Dendritic cells are present in the choroid plexus and in the CSF. NKs and ILC1 have been found in the choroid plexus but in smaller amounts compared with the meninges, while ILC3 is absent and ILC2 abundance increases in an age-dependent way. Perivascular spaces are populated mainly by dendritic cells and PvMΦ (CD163+, CD206+, and Lyve1+). PvMΦ is located between the vascular basement membrane and the glial limitans of the brain parenchyma and is part of the neurovascular unit (NVU), composed of non-fenestrated endothelial cells, pericytes, and astrocyte endfeet.

Apart from CAMs, the brain also harbors resident dendritic cells (DCs) and innate lymphoid cells (ILCs), as illustrated in Figure 1. Although DCs and ILCs are critical members of the CNS immune landscape, this review focuses mostly on CAMs, with some general references to the other cell types.

### 3.2. Microglia

Microglia are a core cell type of the CAM system and keepers of CNS homeostasis. Microglia patrol the CNS to detect pathogens or tissue injury [33,34] and are deeply involved in synaptic pruning during neurodevelopment [46,47]. Microglia contribute to synaptic remodeling following tissue damage [48,49] and modulate neuronal survival and neurogenesis through the secretion of growth factors such as IGF-1 [50], TGFβ [51], m-CSF [52], ARG-1 [53], and BDNF [54,55]. Other mechanisms through which microglia influence the neurogenesis of the adult hippocampus involve two phagocytic pathways: the purinergic receptor P2Y12 and the tyrosine kinases of the TAM family Mer tyrosine kinase (MerTK)/Axl. The study of Diaz-Aparicio et al. demonstrates that neurogenesis is transiently increased in mice in which MerTK was conditionally downregulated, while it is disrupted in mice chronically deficient for the two phagocytic pathways. Interestingly, the authors of this study found that the secretome of phagocytic microglia limits the production of new neurons both in vivo and in vitro. They suggest that microglia can operate as a local sensor of cell death, modulating the balance between proliferation and survival in the neurogenic niche through the phagocytosis secretome and supporting the long-term maintenance of adult hippocampal neurogenesis [56]. Microglia seem to exert different functions that appear to be region-specific either in the adult or in the developing brain [57,58]. Indeed, it has been reported that neonatal microglia, but not healthy adult microglia, exhibit a unique myelinogenic and neurogenic phenotype [59]. Neonatal CD11c^+^ microglia cells predominate in primary myelinating areas of the developing brain and express a transcriptional signature that can sustain neuronal and glial cell survival, migration, and differentiation. Furthermore, these specific microglia cells are the primary source of IGF-1, a peptide hormone that is essential for proper neurodevelopment [60]. CD11c^+^ microglia are also found in adult mice during neuroinflammation, although they do not recapitulate what they express in neonatal life [59]. Another key signaling pathway operating in microglia during brain development is the Triggering receptor expressed in the myeloid cells 2 (Trem2) pathway, which controls the bioenergetic profile of pyramidal neurons of the hippocampus [61]. Trem2 is a myeloid cell-specific gene that is only expressed by microglia in the brain [62] and is essential for microglia-mediated synaptic refinement. In the absence of Trem2, developing neurons in the hippocampal Cornus Ammonis (CA)1, but not in the CA3 subfield, display a compromised energetic metabolism and cause a transcriptional rearrangement of hippocampal pyramidal neurons at birth, which is followed by altered synapses and circuit maturation [61].

At steady-state, microglia cell morphology features a small cell body with a highly branched ramified morphology. Key phenotypic markers expressed by resting cells are TMEM119, SALL1, CX3CR1, P2Y12, MerTK, and Iba1 [63].

During neuroinflammation, tissue injury, or neurodegeneration, microglia undergo activation through a highly dynamic transition process that involves many intermediate states [64]. Different activation states are reflected by an altered cellular morphology. Indeed, soon after their activation, microglial cell bodies increase in size, and the cellular processes become shorter and thicker [65]. Apart from morphological rearrangements, the activation of microglia includes a series of functional alterations, such as the downregulation of homeostatic genes [66] and metabolic reprogramming [67]. Among key homeostatic genes, *Trem2* and *Apoe* are involved, respectively, in phagocytosis and chemotaxis, as well as lipid metabolism following microglial uptake of myelin lipid debris [68]. Reactive microglia also upregulate the expression of MHC-II, CD206, CD86, and CD16 [69]. One of the main pathways of activation of innate immune cells, including microglia, is NF-κB. At homeostasis, NF-kB is sequestered in the cytoplasm through interacting with inhibitors of κB (IκB): IκBα, IκBβ, and p100 [70]. Upon stimulatory signals, IKKβ phosphorylates IκB and enables NF-κB to enter the nucleus [71]. Interestingly, NF-kB has been implicated in multiple phenotypes of both homeostatic and reactive microglia [72]. On the one hand, under physiological conditions, the microglial NF-kB/IKKβ pathway regulates hippocampal synaptic plasticity [73]. On the other hand, this pathway in the EAE model can exert either protective or detrimental functions, which depend on the time frame in which the pathway starts to be activated. While at the early stage of the disease, microglial activation through NF-kB may accelerate the onset of the disease, at later time points, NF-kB activation in microglia and macrophages protects against CNS infiltration by peripheral immune cells [74]. Finally, depletion of IKKβ in myeloid cells results in enhanced neuronal long-term potentiation in EAE, suggesting that brain cognitive abilities may also be regulated by the approved treatments blocking NF-κB activity in MS [75].

### 3.3. Border-Associated Macrophages (BAMs)

Given their spatial localization, BAMs are also called “non-parenchymal macrophages;” this definition per se discriminates these cells from parenchymal microglia. Indeed, BAMs dwell at the interface between the brain and the periphery and are classified mainly by their regionalization. BAMs are close to CNS borders and include macrophages of the perivascular space (perivascular macrophages, PvMΦ), the choroid plexus (cpMΦ), and cells located within the meninges (meningeal macrophages, MnMΦ) [76]. Although BAMs have been recently deeply characterized and classified [77], very little is known about the specific functions exerted by these cells in physiological and pathological conditions. The development of single-cell technologies allowed the identification of specific markers for each BAM subtype. In general, BAM subsets can be distinguished by the differential expression of the following genes: CD38, Lyve1, MHC-II, and CCR2. Blood circulating CCR2^+^ myeloid cells likely represent fresh hematopoietic myeloid progenitors that can replace the BAMs accumulating in the choroid plexus [78] and dura mater. CD38^+^ or Lyve1^+^ BAMs are normally CX3CR1^low/negative^, while MHC-II^+^ BAM subsets are CX3CR1^high^. Lyve1^+^MHC-II^+^ BAMs are enriched in the pia mater, perivascular spaces, and choroid plexus while being absent in the dura mater, a region rich in Lyve1 negative MHC-II^+^ BAMs [77].

CAM and BAM subsets can be clearly phenotypically distinguished in the healthy brain. However, upon activation (i.e., in the pathological brain), these cells become indistinguishable because they express similar molecular markers [77]. Thus, similar to microglia, BAMs are highly plastic cells that can change their gene expression patterns in the disease state [45]. In contrast to monocyte-derived cells and DCs, which increase in numbers during EAE, BAMs decrease, lose their heterogeneity, and almost exclusively co-express CD38 and MHC-II [77]. CAMs express over a thousand receptors and likely respond to hundreds, if not thousands, of molecules that can drive changes in their functionality. Being a member of the innate immunity family, they sense the environment through extra- and intra-cellular pathogen recognition receptors (PRRs), recognizing damage-associated molecular patterns released in the CNS in response to cellular injury and death [79]. Reactive microglia and macrophages also produce inflammatory cytokines such as TNFα, IL-6, IL-1β, and IL-23, leading to sustained immune activation [80].

MnMΦ: The meninges are composed of three layers of membranes comprising the dura mater (the outer-most layer), the arachnoid mater, and the pia mater (inner layers), which surrounds the brain and the spinal cord [81]. The meninges of the healthy brain are populated by BAMs, which can be distinguished mainly in subdural and dural macrophages. Subdural macrophages are characterized by the following pattern of expression: MHC-II ^low^, Egfl7^+^, and Lyve1^+^, while dural macrophages are either MHC-II high or low [82,83]. Recent findings demonstrate that the dural sinus is a site of immune surveillance where antigen-presenting cells (APCs), most likely dural MHC-II ^high^ BAMs, capture brain-derived antigens and interact with T cells [84]. Moreover, the study of Rebejac et al. [85] demonstrates that meningeal macrophages are crucial in case of viral infection. BAMs featuring MHC-II^high^ are essential to counteract peripheral lymphocytic choriomeningitis viruses that lead to a transient infection and activation of the meningeal cell populations [85].

CpMΦ: The primary function of the choroid plexus is the secretion and modulation of CSF, as well as waste and metabolite removal [86]. BAMs are the largest class of immune cells present in the choroid plexus and are predominantly associated with blood vessels [87]. They express Apoe, Ms4a7, and Ms4a6c [88]. As seen for dural BAMs, cpMΦ can also express different levels of MHC-II [82,88], but, contrary to dural BAMs, this distinction does not match with distinct cellular clusters with specific functions; rather, it seems to be age-dependent, suggesting a maturation-dependent shift from cells with low levels of MHC-II to cells with high levels of MHC-II [88]. Although additional studies are needed to clarify the function of these cells, the transcriptional profile of CpMΦ strongly suggests that they are involved in lipid metabolism, phagocytosis, antigen presentation, and immune responses [82]. They are most probably involved also in regulating CSF homeostasis since mice lacking CpMΦ display enlarged ventricles and hydrocephaly [89]. Further evidence comes from a recent study using CellChat analysis [90], reporting that CpMΦ induces pathogenic CSF production in a hydrocephalus mouse model [91].

PvMΦ: they reside in the perivascular space, between the vascular basement membrane and the glial limitans of the brain parenchyma. Together with non-fenestrated endothelial cells, mural cells, and astrocytic end feet, they compose the neurovascular unit (NVU), the minimal functional unit of the BBB, which has the important role of restricting the entry of neurotoxic components, pathogens, or peripheral immune cells into the brain [92]. CD163, CD206, and Lyve1 [93] are hallmarks of PvMΦ. PvMΦ is reported to have a region-specific distribution, with the highest abundance in the olfactory bulb and hippocampus [92]. PvMΦ is likely to play key functions in the maintenance of the BBB integrity, patrolling the entry of macromolecules into the brain [93,94]. Indeed, several results obtained from their experimental ablation using pharmacological or genetic tools indicate that these BAMs can contribute to the fine-tuning of cerebral blood flow and regulation of hypercapnia-induced vasodilation [95]. Moreover, PvMΦ can establish purinergic contacts with cells in the NVU in both mice and humans [84,85,96,97] and maintain vascular integrity by engulfing foreign macromolecules, as shown with the 10 to 70 kDa dextran [98]. They also regulate BBB permeability by regulating the expression of tight junctions through pigment epithelium-derived factors, both in vitro and in vivo [99]. PvMΦ is also implicated in pathological conditions, such as cerebral malaria [100] and diet-induced hypothalamic inflammation [101,102]. Furthermore, PvMΦ is an important player in CNS glucose metabolism and hypothalamic–pituitary–adrenal axis regulation [103]. The PvMΦ population was found to contribute also to vascular leakage and granulocyte recruitment in the acute phase of stroke [104], as well as to neurovascular alterations in Alzheimer’s disease [105]. Lastly, PvMΦs are linked to hypertension that occurs following systemic inflammation and brain ischemia. Indeed, the PvMΦ cell population is modulated by circulating inflammatory cytokines such as IL-1β and contributes to the development of hypertension through sympathoexcitation [103]. By releasing soluble signals, such as cytokines, these cells may also be involved in angiogenesis and vascular remodeling in both physiological and pathological conditions [106].

### 3.4. Dendritic Cells

DCs are highly specialized professional APCs expressing MHC-II along with costimulatory molecules. Since DCs activate T and B cells, they represent a bridge between innate and adaptive immune cells [107]. At the same time, DCs express a plethora of PRRs, such as Toll-like receptors (TLRs), which lead these cells to a massive secretion of pro-inflammatory cytokines and type I interferon [108]. Plasmacytoid DCs (pDCs) are unconventional DCs that do not present antigens but produce large amounts of type I interferon in response to pathogen detection [109]. DCs represent 1% of total immune cells in the brain and are also detectable in the CSF [110,111,112]. Brain DCs contribute to tissue patrolling and neuroinflammation [113]. In physiological conditions, DCs localize at the level of the choroid plexus, meninges, and perivascular spaces while they invade the parenchyma in response to inflammatory conditions [114]. Therefore, CNS-resident DCs are in an optimal position to interact with infiltrating T cells as they are close to CNS entry points. We want to underscore the presence of conventional DCs (cDCs) in dural sinuses, allowing the presentation of antigens coming from the CSF [84,115]. CNS-DCs are divided into three main subsets, corresponding to cDCs1, cDC2, and pDCs, which can be distinguished by the differential expression of several markers, such as CD11b, CD172, and CD24. The cDC1 cluster is a homogenous cell population identified as CD11b^low^ CD172^low^ CD24^+^ CD135^high^ CD117^+^. The cDC2 subset is slightly more abundant than the cDC1 subset [66]. These cells express high levels of CD11b and CD172 and can be further distinguished into CD24^+^CD206^+^ cDC2, CD24^neg^ CD64^+/low^ CD206^−/low^ cDC2, CD135^high^ cDC2, and PDL1^+^ cDC2. pDCs are defined as Ly6C^+^ B220^+^ Siglec-H^+^ [66].

### 3.5. Innate Lymphoid Cells

Even if it is out of the scope of this article, we mention the ILCs that are part of the CNS resident innate immune populations below. ILCs are the innate counterparts of T cells, but they lack antigen receptor rearrangement. The ILC family comprises natural killer (NK) cells, Lymphoid Tissue Inducer (LTi) cells, ILC1, ILC2, and ILC3. NK cells are considered the innate counterpart of CD8^+^ T lymphocytes, while the other subsets share characteristics of helper CD4^+^ T cells. More in detail, Th1, 2, and 17 correspond to ILC1, 2, and 3. In homeostatic conditions, the CNS parenchyma is almost devoid of ILCs, but they are found in regions belonging to the non-parenchymal CNS [116].

NK cells and ILC1 are both defined by the expression of NK1.1^+^ NKp46^+^, while they are differentially expressing CD49b^+^ (ILC1) versus CD49a (NK), the transcription factors Eomes and T-bet (NKs) versus T-bet exclusively (ILC1) [117]. Both NKs and ILC1 produce mainly IFNγ. While NKs are cytotoxic, ILC1s are generally non-cytotoxic due to the lower expression of perforin and granzyme B [118]. NKs and ILC1 have been found in the meninges and, in smaller amounts, in the choroid plexus.

The ILC2 population protects the brain against helminth parasites that may infect the CNS [119]. ILC2s communicate with neuronal populations through the transmembrane receptor RET (REarranged during Transfection), a tyrosine kinase that is activated by the neuronal glial-derived neurotrophic factor (GDNF) and leads to the secretion of IL-5 and IL-13 [120]. In the healthy brain, ILC2 cells populate mainly the dural meninges while they are absent in the leptomeninges (arachnoid mater and pia mater) [121].

In the choroid plexus, the abundance of ILC2s seems to be age-dependent: few ILC2s are present in a healthy young brain, while this number increases with aging, most probably because once they have infiltrated the plexus do not re-enter into the blood circulation and accumulate locally [121]. Another working hypothesis explaining the accumulation of ILC2s over time involves their transcriptional plasticity, enabling the differentiation of NKs and ILC1s into ILC2s [122].

ILC3 cells depend on the transcription factor RORγt and are generally divided into two main cell subsets, NCR^−^ and NCR^+^ ILC3s. The NCR^−^ cell population includes LTi cells generated before birth and LTi-like cells generated after birth [123]. Heterogeneous LTi/LTi-like cells and NCR^+^ ILC3s have been found in the meninges [124].

### 3.6. Ontogeny of the CNS Resident Myeloid Compartment

Microglia and BAMs are long-lived and self-renewing cells that mostly originate from embryonic progenitors in the prenatal yolk sac that colonize the brain during development upon migration [125]. Microglia, MnMΦ, and PvMΦ do not rely on circulating bone marrow-derived hematopoietic progenitors to refill their population, while CpMΦ are partially replenished by circulating monocytes [82]. Although meningeal macrophages were supposed to be independent of circulating precursors [78], dural macrophages are gradually refilled by monocytes over time, probably due to a more permissive barrier state compared with the leptomeninges [82]. Indeed, skull bone marrow monocytes can enter the dura via specialized channels and enrich the local immune landscape in case of neuroinflammation [126]. Interestingly, the study of Bennett et al. [127] shows that microglial identity is shaped by both ontogeny and environmental signals and that the surrounding nervous tissue is pivotal to sustaining homeostatic gene expression in microglia [127].

DCs arise in the bone marrow from a common DC precursor called pre-DC cells and expand in response to the FMS-like receptor tyrosine kinase 3 (FLT3) ligand [128,129]. Importantly, resident DCs are clearly distinguished from peripheral inflammatory monocyte-derived DCs [130].

## 4. CNS Innate Immunity Contribution to Neuroinflammation

**Figure 2:**
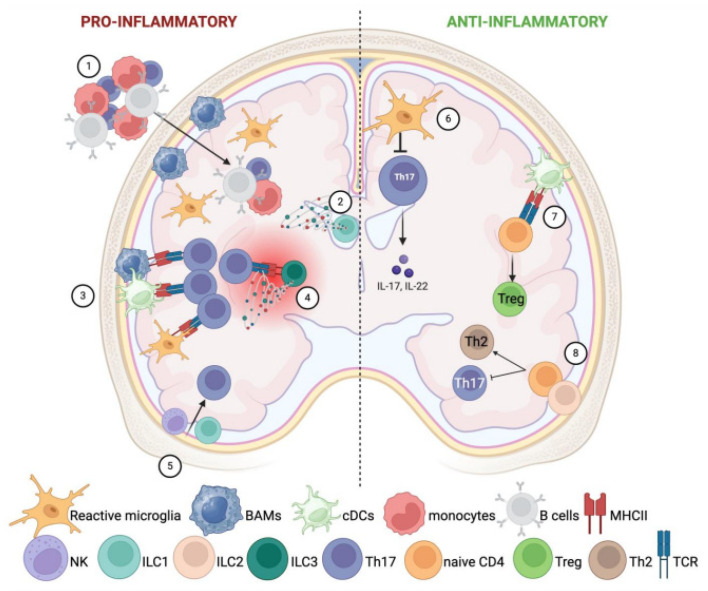
**Involvement of CNS resident innate immune cells in neuroinflammation.** CNS resident innate immune cells can both boost and fight against neuroinflammation. The left side of the image represents their pro-inflammatory potential (1–5), while on the right side, it is depicted as their anti-inflammatory role (6–8). Left side: (1) Microglia and meningeal macrophages recruit peripheral inflammatory cells into the CNS. (2) ILC1s in the choroid plexus favor the entry of peripheral inflammatory cells through the expression of IFN-γ and TNF-α. (3) Microglia, BAMs, and resident cDCs foster T-cell activation through overexpression of MHC-II and co-stimulatory molecules. (4) ILC3s act as APCs to autoimmune T cells in focal lesions of the CNS parenchyma. Furthermore, accumulated ILC3s release pro-inflammatory cytokines such as IFN-γ, IL-17, and GM-CSF, which boost chronic inflammation. (5) NK and ILC1 favor the recruitment of inflammatory Th17. Right side: (6) Microglia can phagocytose and kill CNS-infiltrating Th17 cells, counteracting inflammation. (7) After the disease onset, cDCs prime the development of regulatory T cells instead of Th17. (8) ILC2 switch the differentiation of CD4^+^ T cells into Th2, at the expense of Th17 activation.

As introduced in Chapter 1, neuroinflammation is one of the driving features of MS, modeling disease pathogenesis. In the earlier MS phase, neuroinflammation is characterized by peripheral inflammatory cell waves that infiltrate the CNS, given the increased BBB permeability [131]. Although the initial inflammatory phase is composed mainly of activated autoreactive T lymphocytes, Th1 and Th17, CNS innate immune cells are required for the initiation of the disease. As illustrated in Figure 2, they can both boost and fight against neuroinflammation. Indeed, on the one hand, microglia can phagocytose and kill CNS-infiltrating Th17 cells [132], while on the other hand, it has been reported that microglia and meningeal macrophages are essential in the EAE onset since depleting them with an inhibitor of colony-stimulating factor 1 receptor provoked around five days of delay in EAE onset and a decrease in inflammatory cells into the CNS [133,134]. BAMs also contribute to neuroinflammation by recruiting immune cells to the sites of inflammation and scavenging debris [135,136].

Upon activation, BAMs upregulate the expression of key molecules for antigen presentation, such as MHC-II and CD44 [137]; such activation has been associated with their ability to foster T cell activation after the initial inflammatory phase [77]. Accordingly, the work of Montilla et al. demonstrates the role of microglia and BAMs in antigen presentation during EAE onset [134]. However, other published results support the hypothesis that such a role is mainly played by cDCs and not by BAMs [138,139]. In the study published by Mundt et al., the authors used a combination of high-dimensional single-cell mapping and conditional MHC-II ablation across all CNS APCs to identify DCs as responsible for the reactivation of encephalitogenic T cells in vivo [138]. Along the same lines, the study of Giles et al. demonstrates that cDCs residing in the meninges, brain, and spinal cord undergo a clear expansion inside the parenchyma during neuroinflammation, and the selective depletion of these cDCs leads to a decrease in the number of myelin-primed donor T cells in the CNS, therefore greatly reducing the incidence of clinical EAE [139]. It is still unclear which APC population is predominant in antigen presentation during the onset of EAE, but certainly, innate immune cells are a pivotal driving force for the initial phase of neuroinflammation. The precise mechanisms by which CNS-associated APCs facilitate autoimmune T-cell reactivation are still largely unknown, but one possible explanation is the autophagic pathway. Indeed, transgenic mice bearing the conditional deletion of ATG5 (which belongs to the E3-like complex that catalyzes the lipidation of ATG8 proteins such as LC3B) [140] in DCs are EAE resistant [141,142]. Furthermore, a study by Keller et al. demonstrates that DC-mediated antigen processing is dependent on LC3-associated phagocytosis [143].

During inflammation, CSF-resident DCs infiltrate the inflamed brain and reach the cervical lymph nodes, where they enhance the systemic humoral response against immunogenic myelin antigen [144]. Both resident DCs and peripheral inflammatory monocyte-derived DCs are found at the site of inflammation [139,145], but only CNS resident cDCs are capable of processing immunogenic peptides from larger myelin fragments and activating myelin-specific naive, as well as effector, CD4+ T cells [139]. After the disease onset, DCs decrease their antigen processing potential and prime regulatory T cells [146,147], switching toward an immune-modulatory profile. Indeed, selective depletion of resident DCs can give rise to different phenotypes depending on the disease phase at which the depletion has been performed. In summary, DC depletion can reduce the pathogenicity of EAE [139], but it can also alter the immune tolerance, leading to an excessive inflammatory response [148,149].

Concerning the ILC populations, it has been shown that these cells have subset-specific functions in the frame of neuroinflammation. Depletion of NK and ILC1 through anti-NK1.1 antibody or using Tbx21^−/−^ (encoding T-Bet) or using Tbx21^f/f^ NKp46^Cre+^ mice suppress Th17-mediated neuroinflammation in EAE [150,151]. In addition, ILC1s of the choroid plexus act most probably as gatekeepers for the entry of peripheral inflammatory cells, mainly by the secretion of IFN-γ and TNFα. Both cytokines upregulate the expression of a plethora of trafficking molecules in epithelial cells, such as VCAM1and ICAM1, as well as chemokines, such as CCL2, CCL5, CXCL9, CXCL10, and CX3CL1 [152].

Interestingly, ILC2s have been implicated in gender bias effects observed in MS patients: female patients display a disease onset at a younger age and exhibit a more severe disease course than males. How can ILC2 be linked to this phenotype? A possible answer to this question involves the testosterone that increases IL-33 expression, a cytokine that activates ILC2, which, in turn, increases Th2 responses and limits Th17-dependent demyelination [153].

ILC3s act as APCs to autoimmune T cells in focal lesions of the CNS parenchyma [150,154]. Along the same lines, the deletion of MHC-II^+^ ILC3s substantially ameliorates EAE. On the other hand, the accumulation of ILC3s produces pro-inflammatory cytokines such as IFN-γ, IL-17, and GM-CSF, which are responsible for chronic inflammation [124].

In conclusion, as seen for myeloid cells, ILCs can also have either a beneficial or a detrimental role during neuroinflammation.

## 5. CNS Innate Immunity Contribution to Neurodegeneration

A characteristic feature of MS is the presence of inflammatory T and B lymphocytes, plasma cells, and macrophages in the meninges and the perivascular spaces [155]. Often, these immune cells are organized in large lymphoid-like aggregates and are detectable in patients with short disease duration as well as in patients with PMS [156,157,158]. Lymphoid follicles are associated with severe microglia activation and cortical demyelination. Notably, 40–70% of people with SPMS display these lymphoid structures, which are absent in patients with PPMS. Nonetheless, patients with PPMS display increased meningeal inflammation associated with extensive cortical demyelination and neurite loss, even in the absence of lymphoid follicles [159]. Meningeal inflammation is thought to be one of the leading causes of meningeal-associated cortical lesions and subpial lesions, both prominent in PMS [160]. These lesions are the site of active demyelination, the first cause of neurodegenerative progression.

The only way to stop, or perhaps to reverse disease progression, would be to stimulate active remyelination within the damaged tissue. The broad CAMs family is necessary for remyelination and may have several other neuroprotective roles. Since, following activation, microglia, and macrophages share most of their phenotypical markers, it is difficult to identify the contribution of a specific subset versus another one. Thus, they are mostly considered to behave as a single functional entity. As described for neuroinflammatory mechanisms, in the case of neurodegeneration, both microglia and macrophages can have either pro-inflammatory or anti-inflammatory functions, which depend on the disease stage. On the one hand, they help recruit oligodendrocyte progenitor cells (OPCs) and secrete neurotrophic factors such as IGF-1, which exert pivotal functions for OPC survival and their differentiation into mature myelinating oligodendrocytes [161]. On the other hand, CAMs in early CNS lesions of both MS and preclinical mouse models have a clear pro-inflammatory role and contribute to secreting inflammatory factors such as nitric oxide, TNFα, and IL-1β [162].

CAMs actively phagocyte and clear myelin debris, which is rich in cholesterol. The cholesterol intake is the reason why, along with the process, they acquire a lipid-laden, foamy cellular shape. This cholesterol-driven phenotype causes a switch towards a reparative anti-inflammatory [163,164] identity, which is molecularly explained as follows: myelin breakdown generates lipid and cholesterol metabolites that are internalized by phagocytosis and activate nuclear liver X receptors (LXR), which reverse cholesterol transport and modulate inflammation [165]. At the same time, LXR-mediated cholesterol efflux increases the release of immunomodulatory factors such as IL-10 [166]. Unfortunately, this is not the end of the story. The resulting cholesterol build-up forms crystals that disrupt the lysosomes and activate the inflammasome pathway, restoring the pro-inflammatory phenotype [167,168]. Microglial cholesterol metabolism is regulated by TREM-2. Indeed, it has been shown that lipid droplet formation upon myelin uptake is necessary for triggering a regenerative response, and it is dependent on TREM-2. CAMs deriving from TREM-2-deficient mice are unable to adapt to excess cholesterol exposure and form fewer lipid droplets, thus being more susceptible to cellular stress [169]. Furthermore, Cignarella and colleagues demonstrated that TREM2 is highly expressed on myelin-laden CAMs observed in actively demyelinating lesions in the CNS of subjects with MS. In parallel, subjects with a genetic deficiency of TREM2 display a defect in phagocytic pathways [170].

The importance of CAMs within the MS lesions is underlined by the fact that the newest definition and classification of MS lesions takes into consideration also the number and phenotype of infiltrating CAMs in addition to the demyelination/remyelination state of the lesion [171]. The current guidelines distinguish between active, mixed active–inactive, or inactive lesions [162].

Active lesions, present both in the white and the grey matter, are chronologically the first appearing lesions and are the most frequent lesions found in MS patients with a short disease duration or a diagnosis of RRMS. Along with disease progression, they decrease in frequency. Active lesions are hypercellular and characterized by loss of myelin and a dense infiltration with foamy CAMs, as well as by astrogliosis with increased GFAP expression [172,173]. T cells are also present, but their number is lower than myeloid cells [162,174]. Active lesions contain neurons that are subjected to several stress factors and high levels of oxidative damage that most probably depend on the release of nitric oxide by CAMs in response to the inflammatory environment [175]. Active lesions can be further distinguished into active demyelinating and active post-demyelinating lesions based on the demyelination state. Active and demyelinating lesions include areas with ongoing demyelination. These lesions are characterized by phagocytic macrophage/microglia. Active and post-demyelinating lesions are densely infiltrated by foamy, lipid-containing macrophages/microglia and lack myelin degradation products [162].

Mixed active/inactive lesions are featured by demyelination and by the presence of a hypocellular center almost entirely depleted of CAMs. The center is bordered by a rim of activated CAMs. Moderate T-cell infiltrates are present perivascularly or throughout the lesion center. Hypertrophic astrocytes are also detectable [171]. This type of lesion is observed mostly in patients with PMS or subjects with a disease history of more than 10 years [176]. In these lesions, the thickness of the myeloid rim is highly variable and seems to reflect the speed of lesion evolution. The CAMs can be either foamy or still actively phagocytic [176].

Inactive lesions are the most common lesions observed in PMS; they are extensively demyelinated, have clear borders, and have no ongoing myelin loss. The CAM density is lower or similar to normal white, grey, and deep grey matter of healthy controls. CAMs present in these lesions show a predominantly surveillant ramified morphology; they contain much less degraded myelin products, and some express homeostatic microglia markers such as P2RY12 [15,176,177].

Even though lesions are considered the principal site of neurodegeneration, MS patients also present alterations in the normal-appearing white and grey matter. The density of CAMs is similar to the density that is observed in age-related healthy controls; however, cell reactivity is increased, as shown by a reduced expression of homeostatic markers such as P2RY12 [162].

### 5.1. Dendritic Cells

DCs secrete pro-inflammatory cytokines, which are critical in recruiting immune components to demyelinating sites [178,179]. Mature DCs are found in postmortem brains and spinal cords of MS patients. They are localized in meningeal infiltrates and are often close to proliferating lymphocytes [180], suggesting that meningeal DCs might promote the formation of demyelinating lesions. Another evidence supporting the implication of DCs in neuroinflammation comes from clinical trials with some DMTs. For example, the decrease in relapses observed in MS patients treated with Natalizumab, a recombinant humanized IgG4κ monoclonal antibody that binds to α4-integrin, was associated with a reduction in the DC number in lesions [181].

### 5.2. ILCs

CNS resident ILCs have been implicated in neuroinflammation and demyelination processes. We already described in Chapter 3 that IL-33 orchestrates a beneficial effect through the activation of ILC2 cells [153]. In addition, IL-33 upregulates oligodendrocyte gene expression and myelination through p38/MAPK phosphorylation, promoting remyelination of damaged neurons [182,183], thus creating a functional link that connects ILC2 with reparative processes. Furthermore, meningeal ILC2s exhibit neuroprotective properties by upregulating the expression of CGRP (Calcitonin gene-related peptide I) and other neuroprotective molecules in a mouse model of spinal cord contusion [184,185].

## 6. Conclusions and Therapeutic Perspective

This review aims to discuss the role exerted by tissue-resident innate immune cells in MS pathobiology, considering classical degenerative processes, namely neuroinflammation and the subsequent degeneration process. The cellular and molecular features here described in the frame of MS can also be easily translated to other CNS pathologies in which CAMs are emerging to be active players, such as Alzheimer’s, Parkinson’s, and brain ischemia.

The picture emerging from the recent literature indicates that the interplay between CAMs and the other cell types involved in MS is quite complex. Processes such as inflammation, tissue damage, demyelination, and axonal loss, as well as remyelination and regeneration, can only be accomplished when all the actors involved play their active roles.

MS has been considered for many years a disease mainly driven by T and B lymphocytes, but recent discoveries pointed out a primary role of innate immune cells not only in priming the adaptive responses but also in leading and maintaining local inflammation, demyelination, and associated neurodegeneration.

Whether neuroinflammation or neurodegeneration comes first, to prevent disease progression and promote tissue repair, MS therapies should be active on both processes and consider the role of innate mediators, including microglia, macrophages, DCs, and ILCs.

Although therapeutics solely targeting BAMs are still missing, studies have found that conventional DMTs might exert their neuroprotective effects by acting through activated microglia [186]. Furthermore, several new compounds that are currently at the pre-clinical stage could target CAMs, such as ethyl pyruvate. This compound reduces the expression of high-mobility group box 1 (HMGB1) in activated myeloid cells, inhibiting their pro-inflammatory potential and protecting against EAE [187].

To highlight the importance of innate immune cells as a central functional hub in MS pathogenesis, one of the most promising lines of research is focused on pharmacological inhibitors of Bruton’s tyrosine kinase (BTK), an enzyme expressed in B lymphocytes and myeloid cells, including microglia. Several phase 2 clinical trials on BTK inhibitors resulted in a reduction in acute inflammation, combined with CNS immune modulation [188,189,190]. As CAMs subset can phagocytose myelin, lipid profiles could be a cost-effective predictor of disease progression in MS patients [191]. Similarly, it has been shown that there is a functional association between dyslipidemia and brain atrophy in MS. Therefore, lipids and lipoprotein could be future targets for therapeutic intervention in MS. Moreover, given the importance of cholesterol efflux after myelin-debris accumulation in CNS resident myeloid cells, it has also been demonstrated in the cuprizone preclinical model that treatment with a new TREM2 agonistic antibody promotes the clearance of myelin debris, increased density of oligodendrocyte precursors in the demyelinated areas, as well as the formation of mature oligodendrocytes, enhancing remyelination and axonal integrity [170].

Moreover, specific drugs acting on the innate system components may alleviate some side effects that are associated with classical DMTs and help regain proper CNS immunomodulation. For instance, Natalizumab prevents the infiltration of leukocytes into the CNS [192]. However, the strong immunosuppression declined the surveillance ability of the local immune system, potentially increasing the risk of progressive multifocal encephalopathy. The combined use of agonists for the innate receptor TLR3 could re-establish CNS immune surveillance in the EAE, restoring a proper immune balance and reducing the risk of encephalopathy [193].

To sum up, therapeutic interventions blocking the pro-inflammatory effects of CAMs during disease progression while preserving their anti-inflammatory functions have achieved great success [186]. However, as infiltrating inflammatory monocytes and resident microglia contribute differentially to the disease pathophysiology, it is necessary to develop finely targeted strategies able to discriminate among the different myeloid cells and their activation state, which is dynamic over time and space.

Concerning resident ILCs, it has been shown that some drugs developed against Th1 and Th17 can also be effective on innate lymphoid populations [194]. As an example, immunosuppressant DMTs such as dimethyl fumarate (DMF), Natalizumab, Fingolimod (FTY720—acting on S1P receptors1), and Daclizumab (monoclonal antibody against CD25) target also NK cells [195]. Furthermore, Daclizumab and Fingolimod show an additional effect on other members of the ILC family: Daclizumab expands immunoregulatory CD56^bright^ NK cells in peripheral blood, and CSF [196,197], decreases circulating RORγt^+^ ILCs (normally increased in MS patients versus healthy controls [198]) and shifts the LTi phenotype towards CD56^bright^ NKs [199]. Fingolimod targets NK cells through their expression of S1PR1 and S1PR5; consequently, NK cells’ egress from the lymph nodes is compromised [200]. However, the overall effect of Fingolimod on NK cells is still unclear, given that independent studies reported a phenotypic alteration in the long-term treatment, with increased frequency of circulating CD56^dim^ mature NK cells and decreased amount of CD56^bright^ and CD127^+^ ILCs [201,202]. A study investigating whether ILC subsets also express S1PR1 found that ILC2 expresses S1PR1 upon activation [203]. In line with this finding, a comparison between untreated MS patients and patients under Fingolimod treatment showed that the total number of ILCs in peripheral blood was reduced [200], most probably because Fingolimod blocks ILC2 migration from the gut during an inflammatory state, as seen in mice [203]. Cladribine, too, seems to modulate circulating ILCs. Cladribine acts as a nucleoside analog of deoxyadenosine, inhibiting DNA synthesis and repair and resulting in cell apoptosis [204]. Cladribine has an immunomodulatory role [205]. The study by Aglas-Leitner et colleagues analyzes the effect of Cladribine on blood circulating ILCs in MS patients over time and reported that Cladribine reduces the majority of ILC, but not MS-inhibitory CD56^bright^ NK cells, ILC2, and CD38^+^ NK cells [206]. Although our focus is to underline potential therapeutic mechanisms of action rather than giving an overview of the state-of-art therapeutic approaches, we should remind the reader that some of the DMTs just cited showed some safety issues in the patients. Daclizumab was suspended and withdrawn in 2018 after causing serious inflammatory brain disorders in 12 patients worldwide [207]. Independent reports also underlined cases of tumefactive demyelination in patients after Fingolimod withdrawal or during Fingolimod treatment [208,209,210,211].

In conclusion, when characterizing conventional and new DMTs, it is pivotal to take into account also their potential effect on systemic and CNS resident innate immune cell populations. Lastly, we underline here that the same immune cell subset can have both a beneficial and a detrimental role in the frame of lifelong disease as MS is, and such differences may depend mostly on the disease phase.

## 7. Highlights

- The CNS immune landscape is plastic and multifaceted. Apart from brain parenchyma, it is crucial to consider also immune cell populations residing at the borders: meninges, perivascular space, and choroid plexus.
- The resident CNS innate immune cells are involved in multiple aspects of CNS homeostasis and pathology. Here, we focus on neuroinflammation and neurodegeneration, two interdependent facets of multiple sclerosis, as well as other neurological disorders.
- The same cellular subset can have either a beneficial or detrimental role in the frame of MS, according to the disease phase.
- When analyzing the effect driven by DMTs, it is pivotal to consider both adaptive immunity and innate immunity since they mutually influence each other.

## Abbreviations

APC: Antigen-presenting cells; BAMs: Border-associated macrophages; BBB: Blood-brain barrier; BDNF: Brain-derived neurotrophic factor; CAMs: CNS-associated macrophages; cDCs: conventional dendritic cells; CNS: Central nervous system; CpMΦ: choroid plexus macrophages; CSF: Cerebrospinal fluid; DCs: Dendritic cells; DMTs: Disease-modifying therapies; EAE: Experimental autoimmune encephalomyelitis; GDNF: glial-derived neurotrophic factor;; IFN: Interferon; IGF-1: Insulin-like growth factor 1; ILCs: Innate lymphoid cells; LTi: Lymphoid tissue inducer; LXR: Liver X receptor; M-CSF: Macrophage colony-stimulating factor; MHC-II: Major histocompatibility complex II; MS: Multiple sclerosis; NK: Natural killer; NVU: Neuro-vascular unit; OPCs: Oligodendrocyte progenitor cells; pDCs: plasmacytoid dendritic cells; PMS: Progressive multiple sclerosis; PPMS: Primary-progressive multiple sclerosis; PvMΦ: Perivascular macrophages; RRMS: Relapsing–remitting multiple sclerosis; SPMS: Secondary progressive multiple sclerosis; Th: T helper; TCR: T cell receptor; TGF-β: Transforming growth factor beta; TLR: Toll-like receptor; TNF-α: Tumor necrosis factor alpha. Trem2: Triggering receptor expressed on myeloid cells 2.

## References

[1] Walton C., King R., Rechtman L., Kaye W., Leray E., Marrie R.A., Robertson N., La Rocca N., Uitdehaag B., Van Der Mei I. (2020). Rising prevalence of multiple sclerosis worldwide: Insights from the Atlas of MS, third edition. Mult. Scler. J..

[2] Oh J., Vidal-Jordana A., Montalban X. (2018). Multiple sclerosis: Clinical aspects. Curr. Opin. Neurol..

[3] McGinley M.P., Goldschmidt C.H., Rae-Grant A.D. (2021). Diagnosis and Treatment of Multiple Sclerosis. JAMA.

[4] Aloisi F., Giovannoni G., Salvetti M. (2023). Epstein-Barr virus as a cause of multiple sclerosis: Opportunities for prevention and therapy. Lancet Neurol..

[5] Zhou X., Baumann R., Gao X., Mendoza M., Singh S., Sand I.K., Xia Z., Cox L.M., Chitnis T., Yoon H. (2022). Gut microbiome of multiple sclerosis patients and paired household healthy controls reveal associations with disease risk and course. Cell.

[6] Ascherio A., Munger K.L., White R., Köchert K., Simon K.C., Polman C.H., Freedman M.S., Hartung H.-P., Miller D.H., Montalbán X. (2014). Vitamin D as an early predictor of multiple sclerosis activity and progression. JAMA Neurol..

[7] Olsson T., Barcellos L.F., Alfredsson L. (2016). Interactions between genetic, lifestyle and environmental risk factors for multiple sclerosis. Nat. Rev. Neurol..

[8] Lublin F.D. (2014). New Multiple Sclerosis Phenotypic Classification. Eur. Neurol..

[9] Klineova S., Lublin F.D. (2018). Clinical Course of Multiple Sclerosis. Cold Spring Harb. Perspect. Med..

[10] Hauser S.L., Cree B.A. (2020). Treatment of Multiple Sclerosis: A Review. Am. J. Med..

[11] Pozzilli C., Pugliatti M., Vermersch P., Grigoriadis N., Alkhawajah M., Airas L., Oreja-Guevara C. (2022). Diagnosis and treatment of progressive multiple sclerosis: A position paper. Eur. J. Neurol..

[12] Lassmann H. (2019). Pathogenic mechanisms associated with different clinical courses of multiple sclerosis. Front. Immunol..

[13] Baecher-Allan C., Kaskow B.J., Weiner H.L. (2018). Multiple Sclerosis: Mechanisms and Immunotherapy. Neuron.

[14] Babaloo Z., Aliparasti M.R., Babaiea F., Almasi S., Baradaran B., Farhoudi M. (2015). The role of Th17 cells in patients with relapsing-remitting multiple sclerosis: Interleukin-17A and interleukin-17F serum levels. Immunol. Lett..

[15] Haider L., Simeonidou C., Steinberger G., Hametner S., Grigoriadis N., Deretzi G., Kovacs G.G., Kutzelnigg A., Lassmann H., Frischer J.M. (2014). Multiple sclerosis deep grey matter: The relation between demyelination, neurodegeneration, inflammation and iron. J. Neurol. Neurosurg. Psychiatry.

[16] Lublin F.D., Reingold S.C., Cohen J.A., Cutter G.R., Sørensen P.S., Thompson A.J., Wolinsky J.S., Balcer L.J., Banwell B., Barkhof F. (2014). Defining the clinical course of multiple sclerosis: The 2013 revisions. Neurology.

[17] Kuhlmann T., Moccia M., Coetzee T., A Cohen J., Correale J., Graves J., Marrie R.A., Montalban X., Yong V.W., Thompson A.J. (2023). Multiple sclerosis progression: Time for a new mechanism-driven framework. Lancet Neurol..

[18] Mayhew C.N., Singhania R. (2023). A review of protocols for brain organoids and applications for disease modeling. STAR Protoc..

[19] Marangon D., Caporale N., Boccazzi M., Abbracchio M.P., Testa G., Lecca D. (2021). Novel in vitro Experimental Approaches to Study Myelination and Remyelination in the Central Nervous System. Front. Cell. Neurosci..

[20] Slanzi A., Iannoto G., Rossi B., Zenaro E., Constantin G. (2020). In vitro Models of Neurodegenerative Diseases. Front. Cell Dev. Biol..

[21] Dixit A., Savage H.S., Greer J.M. (2023). An appraisal of emerging therapeutic targets for multiple sclerosis derived from current preclinical models. Expert Opin. Ther. Targets.

[22] Glatigny S., Bettelli E. (2018). Experimental Autoimmune Encephalomyelitis (EAE) as Animal Models of Multiple Sclerosis (MS). Cold Spring Harb. Perspect. Med..

[23] Kipp M. (2024). How to Use the Cuprizone Model to Study De- and Remyelination. Int. J. Mol. Sci..

[24] Hart B.A. (2016). Why does multiple sclerosis only affect human primates?. Mult. Scler. J..

[25] Rivers T.M., Sprunt D.H., Berry G.P. (1933). Observations on attempts to produce acute disseminated encephalomyelitis in monkeys. J. Exp. Med..

[26] Dendrou C.A., Fugger L., Friese M.A. (2015). Immunopathology of multiple sclerosis. Nat. Rev. Immunol..

[27] Stromnes I.M., Goverman J.M. (2006). Passive induction of experimental allergic encephalomyelitis. Nat. Protoc..

[28] Zamvil S.S., Steinman L. (1990). The T Lymphocyte in experimental allergic encephalomyelitis. Annu. Rev. Immunol..

[29] Lu C., Pelech S., Zhang H., Bond J., Spach K., Noubade R., Blankenhorn E.P., Teuscher C. (2008). Pertussis toxin induces angiogenesis in brain microvascular endothelial cells. J. Neurosci. Res..

[30] Raphael I., Mahesula S., Purkar A., Black D., Catala A., Gelfond J.A.L., Forsthuber T.G., Haskins W.E. (2014). Microwave & Magnetic (M2) proteomics reveals CNS-specific protein expression waves that precede clinical symptoms of experimental autoimmune encephalomyelitis. Sci. Rep..

[31] Constantinescu C.S., Farooqi N., O’Brien K., Gran B. (2011). Experimental autoimmune encephalomyelitis (EAE) as a model for multiple sclerosis (MS). Br. J. Pharmacol..

[32] Berer K., Gerdes L.A., Cekanaviciute E., Jia X., Xiao L., Xia Z., Liu C., Klotz L., Stauffer U., Baranzini S.E. (2017). Gut microbiota from multiple sclerosis patients enables spontaneous autoimmune encephalomyelitis in mice. Proc. Natl. Acad. Sci. USA.

[33] Leo H., Kipp M. (2022). Remyelination in Multiple Sclerosis: Findings in the Cuprizone Model. Int. J. Mol. Sci..

[34] Zhan J., Mann T., Joost S., Behrangi N., Frank M., Kipp M. (2020). The Cuprizone Model: Dos and Do Nots. Cells.

[35] Fischbach F., Nedelcu J., Leopold P., Zhan J., Clarner T., Nellessen L., Beißel C., van Heuvel Y., Goswami A., Weis J. (2019). Cuprizone-induced graded oligodendrocyte vulnerability is regulated by the transcription factor DNA damage-inducible transcript 3. Glia.

[36] Pasquini L.A., Calatayud C.A., Uña A.L.B., Millet V., Pasquini J.M., Soto E.F. (2007). The Neurotoxic effect of cuprizone on oligodendrocytes depends on the presence of pro-inflammatory cytokines secreted by microglia. Neurochem. Res..

[37] Taraboletti A., Walker T., Avila R., Huang H., Caporoso J., Manandhar E., Leeper T.C., Modarelli D.A., Medicetty S., Shriver L.P. (2017). Cuprizone Intoxication Induces Cell Intrinsic Alterations in Oligodendrocyte Metabolism Independent of Copper Chelation. Biochemistry.

[38] Morgan M.L., Teo W., Hernandez Y., Brideau C., Cummins K., Kuipers H.F., Stys P.K. (2022). Cuprizone-induced Demyelination in Mouse Brain is not due to Depletion of Copper. ASN Neuro.

[39] Hou J., Zhou Y., Cai Z., Terekhova M., Swain A., Andhey P.S., Guimaraes R.M., Antonova A.U., Qiu T., Sviben S. (2023). Transcriptomic atlas and interaction networks of brain cells in mouse CNS demyelination and remyelination. Cell Rep..

[40] Zirngibl M., Assinck P., Sizov A., Caprariello A.V., Plemel J.R. (2022). Oligodendrocyte death and myelin loss in the cuprizone model: An updated overview of the intrinsic and extrinsic causes of cuprizone demyelination. Mol. Neurodegener..

[41] Salvador F., Deramoudt L., Leprêtre F., Figeac M., Guerrier T., Boucher J., Bas M., Journiac N., Peters A., Mars L.T. (2022). A Spontaneous Model of Experimental Autoimmune Encephalomyelitis Provides Evidence of MOG-Specific B Cell Recruitment and Clonal Expansion. Front. Immunol..

[42] Bettelli E., Pagany M., Weiner H.L., Linington C., Sobel R.A., Kuchroo V.K. (2003). Myelin oligodendrocyte glycoprotein–specific T cell receptor transgenic mice develop spontaneous autoimmune optic neuritis. J. Exp. Med..

[43] Pöllinger B., Krishnamoorthy G., Berer K., Lassmann H., Bösl M.R., Dunn R., Domingues H.S., Holz A., Kurschus F.C., Wekerle H. (2009). Spontaneous relapsing-remitting EAE in the SJL/J mouse: MOG-reactive transgenic T cells recruit endogenous MOG-specific B cells. J. Exp. Med..

[44] Prinz M., Masuda T., Wheeler M.A., Quintana F.J. (2021). Microglia and Central Nervous System–Associated Macrophages—From Origin to Disease Modulation. Annu. Rev. Immunol..

[45] Mildenberger W., A Stifter S., Greter M. (2022). Diversity and function of brain-associated macrophages. Curr. Opin. Immunol..

[46] Neniskyte U., Gross C.T. (2017). Errant gardeners: Glial-cell-dependent synaptic pruning and neurodevelopmental disorders. Nat. Rev. Neurosci..

[47] Paolicelli R.C., Bolasco G., Pagani F., Maggi L., Scianni M., Panzanelli P., Giustetto M., Ferreira T.A., Guiducci E., Dumas L. (2011). Synaptic Pruning by Microglia Is Necessary for Normal Brain Development. Science.

[48] Ding X., Wang J., Huang M., Chen Z., Liu J., Zhang Q., Zhang C., Xiang Y., Zen K., Li L. (2021). Loss of microglial SIRPα promotes synaptic pruning in preclinical models of neurodegeneration. Nat. Commun..

[49] Sellgren C.M., Gracias J., Watmuff B., Biag J.D., Thanos J.M., Whittredge P.B., Fu T., Worringer K., Brown H.E., Wang J. (2019). Increased synapse elimination by microglia in schizophrenia patient-derived models of synaptic pruning. Nat. Neurosci..

[50] Myhre C.L., Thygesen C., Villadsen B., Vollerup J., Ilkjær L., Krohn K.T., Grebing M., Zhao S., Khan A.M., Dissing-Olesen L. (2019). Microglia Express Insulin-Like Growth Factor-1 in the Hippocampus of Aged APPswe/PS1ΔE9 Transgenic Mice. Front. Cell. Neurosci..

[51] De Lucia C., Rinchon A., Olmos-Alonso A., Riecken K., Fehse B., Boche D., Perry V.H., Gomez-Nicola D. (2016). Microglia regulate hippocampal neurogenesis during chronic neurodegeneration. Brain Behav. Immun..

[52] Oosterhof N., Kuil L.E., van der Linde H.C., Burm S.M., Berdowski W., van Ijcken W.F., van Swieten J.C., Hol E.M., Verheijen M.H., van Ham T.J. (2018). Colony-Stimulating Factor 1 Receptor (CSF1R) Regulates Microglia Density and Distribution, but Not Microglia Differentiation In Vivo. Cell Rep..

[53] Stratoulias V., Ruiz R., Kanatani S., Osman A.M., Keane L., Armengol J.A., Rodríguez-Moreno A., Murgoci A.-N., García-Domínguez I., Alonso-Bellido I. (2023). ARG1-expressing microglia show a distinct molecular signature and modulate postnatal development and function of the mouse brain. Nat. Neurosci..

[54] Harley S.B.R., Willis E.F., Shaikh S.N., Blackmore D.G., Sah P., Ruitenberg M.J., Bartlett P.F., Vukovic J. (2021). Selective ablation of BDNF from microglia reveals novel roles in Self-renewal and hippocampal neurogenesis. J. Neurosci..

[55] Zhang J., Rong P., Zhang L., He H., Zhou T., Fan Y., Mo L., Zhao Q., Han Y., Li S. (2021). IL4-driven microglia modulate stress resilience through BDNF-dependent neurogenesis. Sci. Adv..

[56] Diaz-Aparicio I., Paris I., Sierra-Torre V., Plaza-Zabala A., Rodríguez-Iglesias N., Márquez-Ropero M., Beccari S., Huguet P., Abiega O., Alberdi E. (2020). Microglia actively remodel adult hippocampal neurogenesis through the phagocytosis secretome. J. Neurosci..

[57] Tan Y.-L., Yuan Y., Tian L. (2020). Microglial regional heterogeneity and its role in the brain. Mol. Psychiatry.

[58] Menassa D.A., Muntslag T.A., Martin-Estebané M., Barry-Carroll L., Chapman M.A., Adorjan I., Tyler T., Turnbull B., Rose-Zerilli M.J., Nicoll J.A. (2022). The spatiotemporal dynamics of microglia across the human lifespan. Dev. Cell.

[59] Wlodarczyk A., Holtman I.R., Krueger M., Yogev N., Bruttger J., Khorooshi R., Benmamar-Badel A., de Boer-Bergsma J.J., Martin N.A., Karram K. (2017). A novel microglial subset plays a key role in myelinogenesis in developing brain. EMBO J..

[60] Rusin D., Becirovic L.V., Lyszczarz G., Krueger M., Benmamar-Badel A., Mathiesen C.V., Schiöth E.S., Lambertsen K.L., Wlodarczyk A. (2024). Microglia-Derived Insulin-like Growth Factor 1 Is Critical for Neurodevelopment. Cells.

[61] Tagliatti E., Desiato G., Mancinelli S., Bizzotto M., Gagliani M.C., Faggiani E., Hernández-Soto R., Cugurra A., Poliseno P., Miotto M. (2023). Trem2 expression in microglia is required to maintain normal neuronal bioenergetics during development. Immunity.

[62] Kiialainen A., Hovanes K., Paloneva J., Kopra O., Peltonen L. (2005). Dap12 and Trem2, molecules involved in innate immunity and neurodegeneration, are co-expressed in the CNS. Neurobiol. Dis..

[63] Kierdorf K., Prinz M. (2017). Microglia in steady state. J. Clin. Investig..

[64] Vidal-Itriago A., Radford R.A.W., Aramideh J.A., Maurel C., Scherer N.M., Don E.K., Lee A., Chung R.S., Graeber M.B., Morsch M. (2022). Microglia morphophysiological diversity and its implications for the CNS. Front. Immunol..

[65] Paolicelli R.C., Sierra A., Stevens B., Tremblay M.-E., Aguzzi A., Ajami B., Amit I., Audinat E., Bechmann I., Bennett M. (2022). Microglia states and nomenclature: A field at its crossroads. Neuron.

[66] Butovsky O., Weiner H.L. (2018). Microglial signatures and their role in health and disease. Nat. Rev. Neurosci..

[67] Yang S., Qin C., Hu Z.-W., Zhou L.-Q., Yu H.-H., Chen M., Bosco D.B., Wang W., Wu L.-J., Tian D.-S. (2021). Microglia reprogram metabolic profiles for phenotype and function changes in central nervous system. Neurobiol. Dis..

[68] Krasemann S., Madore C., Cialic R., Baufeld C., Calcagno N., El Fatimy R., Beckers L., O’Loughlin E., Xu Y., Fanek Z. (2017). The TREM2-APOE Pathway Drives the Transcriptional Phenotype of Dysfunctional Microglia in Neurodegenerative Diseases. Immunity.

[69] Jurga A.M., Paleczna M., Kuter K.Z. (2020). Overview of General and Discriminating Markers of Differential Microglia Phenotypes. Front. Cell. Neurosci..

[70] Savinova O.V., Hoffmann A., Ghosh G. (2009). The Nfkb1 and Nfkb2 Proteins p105 and p100 Function as the Core of High-Molecular-Weight Heterogeneous Complexes. Mol. Cell.

[71] Liu T., Zhang L., Joo D., Sun S.-C. (2017). NF-κB signaling in inflammation. Signal Transduct. Target. Ther..

[72] Dresselhaus E.C., Meffert M.K. (2019). Cellular Specificity of NF-κB Function in the Nervous System. Front. Immunol..

[73] Kyrargyri V., Vega-Flores G., Gruart A., Delgado-García J.M., Probert L. (2015). Differential contributions of microglial and neuronal IKKβ to synaptic plasticity and associative learning in alert behaving mice. Glia.

[74] Lin W., Yue Y., Stone S. (2018). Role of nuclear factor κB in multiple sclerosis and experimental autoimmune encephalomyelitis. Neural Regen. Res..

[75] Avloniti M., Evangelidou M., Gomini M., Loupis T., Emmanouil M., Mitropoulou A., Tselios T., Lassmann H., Gruart A., Delgado-García J.M. (2024). IKKβ deletion from CNS macrophages increases neuronal excitability and accelerates the onset of EAE, while from peripheral macrophages reduces disease severity. J. Neuroinflamm..

[76] Amann L., Masuda T., Prinz M. (2023). Mechanisms of myeloid cell entry to the healthy and diseased central nervous system. Nat. Immunol..

[77] Mrdjen D., Pavlovic A., Hartmann F.J., Schreiner B., Utz S.G., Leung B.P., Lelios I., Heppner  F.L., Kipnis J., Merkler D. (2018). High-Dimensional Single-Cell Mapping of Central Nervous System Immune Cells Reveals Distinct Myeloid Subsets in Health, Aging, and Disease. Immunity.

[78] Goldmann T., Wieghofer P., Jordão M.J.C., Prutek F., Hagemeyer N., Frenzel K., Amann L., Staszewski O., Kierdorf K., Krueger M. (2016). Origin, fate and dynamics of macrophages at central nervous system interfaces. Nat. Immunol..

[79] Ransohoff R.M., Brown M.A. (2012). Innate immunity in the central nervous system. J. Clin. Investig..

[80] Lively S., Schlichter L.C. (2018). Microglia responses to pro-inflammatory stimuli (LPS, IFNγ+TNFα) and reprogramming by resolving cytokines (IL-4, IL-10). Front. Cell Neurosci..

[81] Derk J., Jones H.E., Como C., Pawlikowski B., Siegenthaler J.A. (2021). Living on the Edge of the CNS: Meninges Cell Diversity in Health and Disease. Front. Cell. Neurosci..

[82] Van Hove H., Martens L., Scheyltjens I., De Vlaminck K., Pombo Antunes A.R., De Prijck S., Vandamme N., De Schepper S., Van Isterdael G., Scott C.L. (2019). A single-cell atlas of mouse brain macrophages reveals unique transcriptional identities shaped by ontogeny and tissue environment. Nat. Neurosci..

[83] Su Y., Zheng H., Shi C., Li X., Zhang S., Guo G., Yu W., Zhang S., Hu Z., Yang J. (2023). Meningeal immunity and neurological diseases: New approaches, new insights. J. Neuroinflamm..

[84] Rustenhoven J., Drieu A., Mamuladze T., de Lima K.A., Dykstra T., Wall M., Papadopoulos Z., Kanamori M., Salvador A.F., Baker W. (2021). Functional characterization of the dural sinuses as a neuroimmune interface. Cell.

[85] Rebejac J., Eme-Scolan E., Paroutaud L.A., Kharbouche S., Teleman M., Spinelli L., Gallo E., Roussel-Queval A., Zarubica A., Sansoni A. (2022). Meningeal macrophages protect against viral neuroinfection. Immunity.

[86] Lun M.P., Monuki E.S., Lehtinen M.K. (2015). Development and functions of the choroid plexus–cerebrospinal fluid system. Nat. Rev. Neurosci..

[87] Cui J., Xu H., Lehtinen M.K. (2021). Macrophages on the margin: Choroid plexus immune responses. Trends Neurosci..

[88] Dani N., Herbst R.H., McCabe C., Green G.S., Kaiser K., Head J.P., Cui J., Shipley F.B., Jang A., Dionne D. (2021). A cellular and spatial map of the choroid plexus across brain ventricles and ages. Cell.

[89] Nandi S., Gokhan S., Dai X.-M., Wei S., Enikolopov G., Lin H., Mehler M.F., Stanley E.R. (2012). The CSF-1 receptor ligands IL-34 and CSF-1 exhibit distinct developmental brain expression patterns and regulate neural progenitor cell maintenance and maturation. Dev. Biol..

[90] Jin S., Guerrero-Juarez C.F., Zhang L., Chang I., Ramos R., Kuan C.-H., Myung P., Plikus M.V., Nie Q. (2021). Inference and analysis of cell-cell communication using CellChat. Nat. Commun..

[91] Robert S.M., Reeves B.C., Kiziltug E., Duy P.Q., Karimy J.K., Mansuri M.S., Marlier A., Allington G., Greenberg A.B., DeSpenza T. (2023). The choroid plexus links innate immunity to CSF dysregulation in hydrocephalus. Cell.

[92] Schaeffer S., Iadecola C. (2021). Revisiting the neurovascular unit. Nat. Neurosci..

[93] Zeisel A., Muñoz-Manchado A.B., Codeluppi S., Lönnerberg P., La Manno G., Juréus A., Marques S., Munguba H., He L., Betsholtz C. (2015). Cell types in the mouse cortex and hippocampus revealed by single-cell RNA-seq. Science.

[94] Karam M., Janbon H., Malkinson G., Brunet I. (2022). Heterogeneity and developmental dynamics of LYVE-1 perivascular macrophages distribution in the mouse brain. J. Cereb. Blood Flow Metab..

[95] Császár E., Lénárt N., Cserép C., Környei Z., Fekete R., Pósfai B., Balázsfi D., Hangya B., Schwarcz A.D., Szabadits E. (2022). Microglia modulate blood flow, neurovascular coupling, and hypoperfusion via purinergic actions. J. Exp. Med..

[96] Polfliet M.M.J., Zwijnenburg P.J.G., van Furth A.M., van der Poll T., Döpp E.A., de Lavalette C.R., van Kesteren-Hendrikx E.M.L., van Rooijen N., Dijkstra C.D., van den Berg T.K. (2001). Meningeal and Perivascular Macrophages of the Central Nervous System Play a Protective Role During Bacterial Meningitis. J. Immunol..

[97] Faraco G., Sugiyama Y., Lane D., Garcia-Bonilla L., Chang H., Santisteban M.M., Racchumi G., Murphy M., Van Rooijen N., Anrather J. (2016). Perivascular macrophages mediate the neurovascular and cognitive dysfunction associated with hypertension. J. Clin. Investig..

[98] Willis C., Garwood C., Ray D. (2007). A size selective vascular barrier in the rat area postrema formed by perivascular macrophages and the extracellular matrix. Neuroscience.

[99] Zhang W., Dai M., Fridberger A., Hassan A., DeGagne J., Neng L., Zhang F., He W., Ren T., Trune D. (2012). Perivascular-resident macrophage-like melanocytes in the inner ear are essential for the integrity of the intrastrial fluid–blood barrier. Proc. Natl. Acad. Sci. USA.

[100] Qin J., Lovelace M.D., Mitchell A.J., de Koning-Ward T., Grau G.E., Pai S. (2021). Perivascular macrophages create an intravascular niche for CD8^+^ T cell localisation prior to the onset of fatal experimental cerebral malaria. Clin. Transl. Immunol..

[101] Lee C.H., Kim H.J., Lee Y.-S., Kang G.M., Lim H.S., Lee S.-H., Song D.K., Kwon O., Hwang I., Son M. (2018). Hypothalamic Macrophage Inducible Nitric Oxide Synthase Mediates Obesity-Associated Hypothalamic Inflammation. Cell Rep..

[102] Jais A., Solas M., Backes H., Chaurasia B., Kleinridders A., Theurich S., Mauer J., Steculorum S.M., Hampel B., Goldau J. (2016). Myeloid-Cell-Derived VEGF Maintains Brain Glucose Uptake and Limits Cognitive Impairment in Obesity. Cell.

[103] Mendes N.F., Velloso L.A. (2022). Perivascular macrophages in high-fat diet-induced hypothalamic inflammation. J. Neuroinflamm..

[104] Gerganova G., Riddell A., Miller A.A. (2022). CNS border-associated macrophages in the homeostatic and ischaemic brain. Pharmacol. Ther..

[105] Park L., Uekawa K., Garcia-Bonilla L., Koizumi K., Murphy M., Pistik R., Younkin L., Younkin S., Zhou P., Carlson G. (2017). Brain Perivascular Macrophages Initiate the Neurovascular Dysfunction of Alzheimer Aβ Peptides. Circ. Res..

[106] Iyonaga T., Shinohara K., Mastuura T., Hirooka Y., Tsutsui H. (2019). Brain perivascular macrophages contribute to the development of hypertension in stroke-prone spontaneously hypertensive rats via sympathetic activation. Hypertens. Res..

[107] Cabeza-Cabrerizo M., Cardoso A., Minutti C.M., da Costa M.P., e Sousa C.R. (2021). Dendritic Cells Revisited. Annu. Rev. Immunol..

[108] Greene J.T., Brian B.F., Senevirathne S.E., Freedman T.S. (2021). Regulation of myeloid-cell activation. Curr. Opin. Immunol..

[109] Hornero R.A., Idoyaga J. (2023). Plasmacytoid dendritic cells: A dendritic cell in disguise. Mol. Immunol..

[110] Pashenkov M., Huang Y.-M., Kostulas V., Haglund M., Söderström M., Link H. (2001). Two subsets of dendritic cells are present in human cerebrospinal fluid. Brain.

[111] Longhini A.L.F., von Glehn F., Brandão C.O., de Paula R.F., Pradella F., Moraes A.S., Farias A.S., Oliveira E.C., Quispe-Cabanillas J.G., Abreu C.H. (2011). Plasmacytoid dendritic cells are increased in cerebrospinal fluid of untreated patients during multiple sclerosis relapse. J. Neuroinflamm..

[112] Esaulova E., Cantoni C., Shchukina I., Zaitsev K., Bucelli R.C., Wu G.F., Artyomov M.N., Cross A.H., Edelson B.T. (2020). Single-cell RNA-seq analysis of human CSF microglia and myeloid cells in neuroinflammation. Neurol.-Neuroimmunol. Neuroinflamm..

[113] Mundt S., Greter M., Becher B. (2022). The CNS mononuclear phagocyte system in health and disease. Neuron.

[114] Ludewig P., Gallizioli M., Urra X., Behr S., Brait V.H., Gelderblom M., Magnus T., Planas A.M. (2016). Dendritic cells in brain diseases. Biochim. Biophys. Acta Mol. Basis Dis..

[115] Croese T., Castellani G., Schwartz M. (2021). Immune cell compartmentalization for brain surveillance and protection. Nat. Immunol..

[116] Wang S., van de Pavert S.A. (2022). Innate Lymphoid Cells in the Central Nervous System. Front. Immunol..

[117] Romero-Suárez S., Serrato A.D.R., Bueno R.J., Brunotte-Strecker D., Stehle C., Figueiredo C.A., Hertwig L., Dunay I.R., Romagnani C., Infante-Duarte C. (2019). The Central Nervous System Contains ILC1s That Differ From NK Cells in the Response to Inflammation. Front. Immunol..

[118] Bernink J.H., Peters C.P., Munneke M., te Velde A.A., Meijer S.L., Weijer K., Hreggvidsdottir H.S., Heinsbroek S.E., Legrand N., Buskens C.J. (2013). Human type 1 innate lymphoid cells accumulate in inflamed mucosal tissues. Nat. Immunol..

[119] Herbert D.R., Douglas B., Zullo K. (2019). Group 2 Innate Lymphoid Cells (ILC2): Type 2 Immunity and Helminth Immunity. Int. J. Mol. Sci..

[120] Cardoso F., Wolterink R.G.J.K., Godinho-Silva C., Domingues R.G., Ribeiro H., da Silva J.A., Mahú I., Domingos A.I., Veiga-Fernandes H. (2021). Neuro-mesenchymal units control ILC2 and obesity via a brain–adipose circuit. Nature.

[121] Fung I.T.H., Sankar P., Zhang Y., Robison L.S., Zhao X., D’souza S.S., Salinero A.E., Wang Y., Qian J., Kuentzel M.L. (2020). Activation of group 2 innate lymphoid cells alleviates aging-associated cognitive decline. J. Exp. Med..

[122] Golomb S.M., Guldner I.H., Zhao A., Wang Q., Palakurthi B., Aleksandrovic E.A., Lopez J.A., Lee S.W., Yang K., Zhang S. (2020). Multi-modal Single-Cell Analysis Reveals Brain Immune Landscape Plasticity during Aging and Gut Microbiota Dysbiosis. Cell Rep..

[123] van de Pavert S.A., Vivier E. (2016). Differentiation and function of group 3 innate lymphoid cells, from embryo to adult. Int. Immunol..

[124] Hatfield J.K., Brown M.A. (2015). Group 3 innate lymphoid cells accumulate and exhibit disease-induced activation in the meninges in EAE. Cell. Immunol..

[125] Prinz M., Erny D., Hagemeyer N. (2017). Ontogeny and homeostasis of CNS myeloid cells. Nat. Immunol..

[126] Cugurra A., Mamuladze T., Rustenhoven J., Dykstra T., Beroshvili G., Greenberg Z.J., Baker W., Papadopoulos Z., Drieu A., Blackburn S. (2021). Skull and vertebral bone marrow are myeloid cell reservoirs for the meninges and CNS parenchyma. Science.

[127] Bennett F.C., Bennett M.L., Yaqoob F., Mulinyawe S.B., Grant G.A., Gephart M.H., Plowey E.D., Barres B.A. (2018). A Combination of Ontogeny and CNS Environment Establishes Microglial Identity. Neuron.

[128] Chen B., Zhu L., Yang S., Su W. (2021). Unraveling the Heterogeneity and Ontogeny of Dendritic Cells Using Single-Cell RNA Sequencing. Front. Immunol..

[129] Lin D.S., Tian L., Tomei S., Amann-Zalcenstein D., Baldwin T.M., Weber T.S., Schreuder J., Stonehouse O.J., Rautela J., Huntington N.D. (2021). Single-cell analyses reveal the clonal and molecular aetiology of Flt3L-induced emergency dendritic cell development. Nature.

[130] Eisenbarth S.C. (2019). Dendritic cell subsets in T cell programming: Location dictates function. Nat. Rev. Immunol..

[131] Attfield K.E., Jensen L.T., Kaufmann M., Friese M.A., Fugger L. (2022). The immunology of multiple sclerosis. Nat. Rev. Immunol..

[132] Wasser B., Luchtman D., Löffel J., Robohm K., Birkner K., Stroh A., Vogelaar C.F., Zipp F., Bittner S. (2020). CNS-localized myeloid cells capture living invading T cells during neuroinflammation. J. Exp. Med..

[133] Nissen J.C., Thompson K.K., West B.L., Tsirka S.E. (2018). Csf1R inhibition attenuates experimental autoimmune encephalomyelitis and promotes recovery. Exp. Neurol..

[134] Montilla A., Zabala A., Er-Lukowiak M., Rissiek B., Magnus T., Rodriguez-Iglesias N., Sierra A., Matute C., Domercq M. (2023). Microglia and meningeal macrophages depletion delays the onset of experimental autoimmune encephalomyelitis. Cell Death Dis..

[135] Mato M., Ookawara S., Sakamoto A., Aikawa E., Ogawa T., Mitsuhashi U., Masuzawa T., Suzuki H., Honda M., Yazaki Y. (1996). Involvement of specific macrophage-lineage cells surrounding arterioles in barrier and scavenger function in brain cortex. Proc. Natl. Acad. Sci. USA.

[136] Pedragosa J., Salas-Perdomo A., Gallizioli M., Cugota R., Miró-Mur F., Briansó F., Justicia C., Pérez-Asensio F., Marquez-Kisinousky L., Urra X. (2018). CNS-border associated macrophages respond to acute ischemic stroke attracting granulocytes and promoting vascular leakage. Acta Neuropathol. Commun..

[137] Dong Y., Yong V.W. (2019). When encephalitogenic T cells collaborate with microglia in multiple sclerosis. Nat. Rev. Neurol..

[138] Mundt S., Mrdjen D., Utz S.G., Greter M., Schreiner B., Becher B. (2019). Conventional DCs sample and present myelin antigens in the healthy CNS and allow parenchymal T cell entry to initiate neuroinflammation. Sci. Immunol..

[139] Giles D.A., Duncker P.C., Wilkinson N.M., Washnock-Schmid J.M., Segal B.M. (2018). CNS-resident classical DCs play a critical role in CNS autoimmune disease. J. Clin. Investig..

[140] Hanada T., Noda N.N., Satomi Y., Ichimura Y., Fujioka Y., Takao T., Inagaki F., Ohsumi Y. (2007). The Atg12-Atg5 conjugate has a novel E3-like activity for protein lipidation in autophagy. J. Biol. Chem..

[141] Keller C.W., Sina C., Kotur M.B., Ramelli G., Mundt S., Quast I., Ligeon L.-A., Weber P., Becher B., Münz C. (2017). ATG-dependent phagocytosis in dendritic cells drives myelin-specific CD4 ^+^ T cell pathogenicity during CNS inflammation. Proc. Natl. Acad. Sci. USA.

[142] Lee H.K., Mattei L.M., Steinberg B.E., Alberts P., Lee Y.H., Chervonsky A., Mizushima N., Grinstein S., Iwasaki A. (2010). In Vivo Requirement for Atg5 in Antigen Presentation by Dendritic Cells. Immunity.

[143] Keller C.W., Kotur M.B., Mundt S., Dokalis N., Ligeon L.-A., Shah A.M., Prinz M., Becher B., Münz C., Lünemann J.D. (2020). CYBB/NOX2 in conventional DCs controls T cell encephalitogenicity during neuroinflammation. Autophagy.

[144] Hatterer E., Touret M., Belin M.-F., Honnorat J., Nataf S. (2008). Cerebrospinal Fluid Dendritic Cells Infiltrate the Brain Parenchyma and Target the Cervical Lymph Nodes under Neuroinflammatory Conditions. PLoS ONE.

[145] King I.L., Dickendesher T.L., Segal B.M. (2009). Circulating Ly-6C+ myeloid precursors migrate to the CNS and play a pathogenic role during autoimmune demyelinating disease. Blood.

[146] Miller S.D., McMahon E.J., Schreiner B., Bailey S.L. (2007). Antigen Presentation in the CNS by Myeloid Dendritic Cells Drives Progression of Relapsing Experimental Autoimmune Encephalomyelitis. Ann. N. Y. Acad. Sci..

[147] Bailey S.L., Schreiner B., McMahon E.J., Miller S.D. (2007). CNS myeloid DCs presenting endogenous myelin peptides ’preferentially’ polarize CD4+ TH-17 cells in relapsing EAE. Nat. Immunol..

[148] Krienke C., Kolb L., Diken E., Streuber M., Kirchhoff S., Bukur T., Akilli-Öztürk Ö., Kranz L.M., Berger H., Petschenka J. (2021). A noninflammatory mRNA vaccine for treatment of experimental autoimmune encephalomyelitis. Science.

[149] Gallizioli M., Miró-Mur F., Otxoa-De-Amezaga A., Cugota R., Salas-Perdomo A., Justicia C., Brait V.H., Ruiz-Jaén F., Arbaizar-Rovirosa M., Pedragosa J. (2020). Dendritic Cells and Microglia Have Non-redundant Functions in the Inflamed Brain with Protective Effects of Type 1 cDCs. Cell Rep..

[150] Kwong B., Rua R., Gao Y., Flickinger J., Wang Y., Kruhlak M.J., Zhu J., Vivier E., McGavern D.B., Lazarevic V. (2017). T-bet-dependent NKp46 + innate lymphoid cells regulate the onset of T H 17-induced neuroinflammation. Nat. Immunol..

[151] Hao J., Liu R., Piao W., Zhou Q., Vollmer T.L., Campagnolo D.I., Xiang R., La Cava A., Van Kaer L., Shi F.-D. (2010). Central nervous system (CNS)–resident natural killer cells suppress Th17 responses and CNS autoimmune pathology. J. Exp. Med..

[152] Kunis G., Baruch K., Rosenzweig N., Kertser A., Miller O., Berkutzki T., Schwartz M. (2013). IFN-γ-dependent activation of the brain’s choroid plexus for CNS immune surveillance and repair. Brain.

[153] Russi A.E., Ebel M.E., Yang Y., Brown M.A. (2018). Male-specific IL-33 expression regulates sex-dimorphic EAE susceptibility. Proc. Natl. Acad. Sci. USA.

[154] Grigg J.B., Shanmugavadivu A., Regen T., Parkhurst C.N., Ahmed A., Joseph A.M., Mazzucco M., Gronke K., Diefenbach A., Eberl G. (2021). Antigen-presenting innate lymphoid cells orchestrate neuroinflammation. Nature.

[155] Zhan J., Kipp M., Han W., Kaddatz H. (2021). Ectopic lymphoid follicles in progressive multiple sclerosis: From patients to animal models. Immunology.

[156] Kutzelnigg A., Lucchinetti C.F., Stadelmann C., Brück W., Rauschka H., Bergmann M., Schmidbauer M., Parisi J.E., Lassmann H. (2005). Cortical demyelination and diffuse white matter injury in multiple sclerosis. Brain.

[157] Magliozzi R., Howell O., Vora A., Serafini B., Nicholas R., Puopolo M., Reynolds R., Aloisi F. (2007). Meningeal B-cell follicles in secondary progressive multiple sclerosis associate with early onset of disease and severe cortical pathology. Brain.

[158] Serafini B., Rosicarelli B., Magliozzi R., Stigliano E., Aloisi F. (2004). Detection of ectopic B-cell follicles with germinal centers in the meninges of patients with secondary progressive multiple sclerosis. Brain Pathol..

[159] Choi S.R., Howell O.W., Carassiti D., Magliozzi R., Gveric D., Muraro P.A., Nicholas R., Roncaroli F., Reynolds R. (2012). Meningeal inflammation plays a role in the pathology of primary progressive multiple sclerosis. Brain.

[160] Ransohoff R.M. (2023). Multiple sclerosis: Role of meningeal lymphoid aggregates in progression independent of relapse activity. Trends Immunol..

[161] Hsieh J., Aimone J.B., Kaspar B.K., Kuwabara T., Nakashima K., Gage F.H. (2004). IGF-I instructs multipotent adult neural progenitor cells to become oligodendrocytes. J. Cell Biol..

[162] Kamma E., Lasisi W., Libner C., Ng H.S., Plemel J.R. (2022). Central nervous system macrophages in progressive multiple sclerosis: Relationship to neurodegeneration and therapeutics. J. Neuroinflamm..

[163] Grajchen E., Hendriks J.J.A., Bogie J.F.J. (2018). The physiology of foamy phagocytes in multiple sclerosis. Acta Neuropathol. Commun..

[164] Locatelli G., Theodorou D., Kendirli A., Jordão M.J.C., Staszewski O., Phulphagar K., Cantuti-Castelvetri L., Dagkalis A., Bessis A., Simons M. (2018). Mononuclear phagocytes locally specify and adapt their phenotype in a multiple sclerosis model. Nat. Neurosci..

[165] Bilotta M.T., Petillo S., Santoni A., Cippitelli M. (2020). Liver X Receptors: Regulators of Cholesterol Metabolism, Inflammation, Autoimmunity, and Cancer. Front. Immunol..

[166] Berghoff S.A., Spieth L., Sun T., Hosang L., Schlaphoff L., Depp C., Düking T., Winchenbach J., Neuber J., Ewers D. (2021). Microglia facilitate repair of demyelinated lesions via post-squalene sterol synthesis. Nat. Neurosci..

[167] Ising C., Venegas C., Zhang S., Scheiblich H., Schmidt S.V., Vieira-Saecker A., Schwartz S., Albasset S., McManus R.M., Tejera D. (2019). NLRP3 inflammasome activation drives tau pathology. Nature.

[168] Cantuti-Castelvetri L., Fitzner D., Bosch-Queralt M., Weil M.-T., Su M., Sen P., Ruhwedel T., Mitkovski M., Trendelenburg G., Lütjohann D. (2018). Defective cholesterol clearance limits remyelination in the aged central nervous system. Science.

[169] Gouna G., Klose C., Bosch-Queralt M., Liu L., Gokce O., Schifferer M., Cantuti-Castelvetri L., Simons M. (2021). TREM2-dependent lipid droplet biogenesis in phagocytes is required for remyelination. J. Exp. Med..

[170] Cignarella F., Filipello F., Bollman B., Cantoni C., Locca A., Mikesell R., Manis M., Ibrahim A., Deng L., Benitez B.A. (2020). TREM2 activation on microglia promotes myelin debris clearance and remyelination in a model of multiple sclerosis. Acta Neuropathol..

[171] Kuhlmann T., Ludwin S., Prat A., Antel J., Brück W., Lassmann H. (2017). An updated histological classification system for multiple sclerosis lesions. Acta Neuropathol..

[172] Sofroniew M.V. (2009). Molecular dissection of reactive astrogliosis and glial scar formation. Trends Neurosci..

[173] Pham H., Ramp A.A., Klonis N., Ng S.W., Klopstein A., Ayers M.M., Orian J.M. (2009). The astrocytic response in early experimental autoimmune encephalomyelitis occurs across both the grey and white matter compartments. J. Neuroimmunol..

[174] Heß K., Starost L., Kieran N.W., Thomas C., Vincenten M.C.J., Antel J., Martino G., Huitinga I., Healy L., Kuhlmann T. (2020). Lesion stage-dependent causes for impaired remyelination in MS. Acta Neuropathol..

[175] Haider L., Fischer M.T., Frischer J.M., Bauer J., Höftberger R., Botond G., Esterbauer H., Binder C.J., Witztum J.L., Lassmann H. (2011). Oxidative damage in multiple sclerosis lesions. Brain.

[176] Frischer J.M., Weigand S.D., Guo Y., Kale N., Parisi J.E., Pirko I., Mandrekar J., Bramow S., Metz I., Brück W. (2015). Clinical and pathological insights into the dynamic nature of the white matter multiple sclerosis plaque. Ann. Neurol..

[177] Zrzavy T., Hametner S., Wimmer I., Butovsky O., Weiner H.L., Lassmann H. (2017). Loss of ‘homeostatic’ microglia and patterns of their activation in active multiple sclerosis. Brain.

[178] Bando Y. (2019). Roads to Formation of Normal Myelin Structure and Pathological Myelin Structure. Adv. Exp. Med. Biol..

[179] Wagner C.A., Roqué P.J., Goverman J.M. (2019). Pathogenic T cell cytokines in multiple sclerosis. J. Exp. Med..

[180] Howell O.W., Reeves C.A., Nicholas R., Carassiti D., Radotra B., Gentleman S.M., Serafini B., Aloisi F., Roncaroli F., Magliozzi R. (2011). Meningeal inflammation is widespread and linked to cortical pathology in multiple sclerosis. Brain.

[181] Häusler D., Akgün K., Stork L., Lassmann H., Ziemssen T., Brück W., Metz I. (2021). CNS inflammation after natalizumab therapy for multiple sclerosis: A retrospective histopathological and CSF cohort study. Brain Pathol..

[182] Yeung S.S.-H., Ho Y.-S., Chang R.C.-C. (2021). The role of meningeal populations of type II innate lymphoid cells in modulating neuroinflammation in neurodegenerative diseases. Exp. Mol. Med..

[183] Natarajan C., Yao S.-Y., Sriram S. (2016). TLR3 Agonist Poly-IC Induces IL-33 and Promotes Myelin Repair. PLoS ONE.

[184] Si Y., Zhang Y., Zuloaga K., Yang Q. (2023). The role of innate lymphocytes in regulating brain and cognitive function. Neurobiol. Dis..

[185] Gadani S.P., Smirnov I., Wiltbank A.T., Overall C.C., Kipnis J. (2017). Characterization of meningeal type 2 innate lymphocytes and their response to CNS injury. J. Exp. Med..

[186] Wang J., Wang J., Wang J., Yang B., Weng Q., He Q. (2019). Targeting microglia and macrophages: A potential treatment strategy for multiple sclerosis. Front. Pharmacol..

[187] Djedović N., Stanisavljevic S., Jevtić B., Momčilović M., Lavrnja I., Miljković D. (2017). Anti-encephalitogenic effects of ethyl pyruvate are reflected in the central nervous system and the gut. Biomed. Pharmacother..

[188] Krämer J., Bar-Or A., Turner T.J., Wiendl H. (2023). Bruton tyrosine kinase inhibitors for multiple sclerosis. Nat. Rev. Neurol..

[189] Vermersch P., Arnold D.L., Wolinsky J., Havrdova E.K., Kinkolykh A., Hyvert Y., Tomic D., Montalban X. (2023). MRI and Clinical Outcomes of Evobrutinib, a Bruton’s Tyrosine Kinase Inhibitor, in Relapsing Multiple Sclerosis Over 2.5 Years of the Open-Label Extension to a Phase 2 Trial. Mult. Scler. Relat. Disord..

[190] Reich D.S., Arnold D.L., Vermersch P., Bar-Or A., Fox R.J., Matta A., Turner T., Wallström E., Zhang X., Mareš M. (2021). Safety and efficacy of tolebrutinib, an oral brain-penetrant BTK inhibitor, in relapsing multiple sclerosis: A phase 2b, randomised, double-blind, placebo-controlled trial. Lancet Neurol..

[191] Zoubi S.A., Esam H., Elzawi E. (2023). Impact of Dyslipidemia on Progression of Multiple Sclerosis. Mult. Scler. Relat. Disord..

[192] Rudick R., Polman C., Clifford D., Miller D., Steinman L. (2013). Natalizumab. JAMA Neurol..

[193] Hussain R.Z., Cravens P.C., Doelger R., Dentel B., Herndon E., Loof N., Tsai P., Okuda D.T., Racke M.K., Stüve O. (2018). TLR3 agonism re-establishes CNS immune competence during α4-integrin deficiency. Ann. Clin. Transl. Neurol..

[194] Hassanabadi N.S., Broux B., Marinović S., Gotthardt D. (2022). Innate Lymphoid Cells—Neglected Players in Multiple Sclerosis. Front. Immunol..

[195] Gross C.C., Schulte-Mecklenbeck A., Wiendl H., Marcenaro E., de Rosbo N.K., Uccelli A., Laroni A. (2016). Regulatory Functions of Natural Killer Cells in Multiple Sclerosis. Front. Immunol..

[196] Skarica M., Eckstein C., Whartenby K.A., Calabresi P.A. (2011). Novel mechanisms of immune modulation of natalizumab in multiple sclerosis patients. J. Neuroimmunol..

[197] Gross C.C., Schulte-Mecklenbeck A., Rünzi A., Kuhlmann T., Posevitz-Fejfár A., Schwab N., Schneider-Hohendorf T., Herich S., Held K., Konjević M. (2016). Impaired NK-mediated regulation of T-cell activity in multiple sclerosis is reconstituted by IL-2 receptor modulation. Proc. Natl. Acad. Sci. USA.

[198] Gross C.C., Schulte-Mecklenbeck A., Hanning U., Posevitz-Fejfár A., Korsukewitz C., Schwab N., Meuth S.G., Wiendl H., Klotz L. (2017). Distinct pattern of lesion distribution in multiple sclerosis is associated with different circulating T-helper and helper-like innate lymphoid cell subsets. Mult. Scler. J..

[199] Perry J.S.A., Han S., Xu Q., Herman M.L., Kennedy L.B., Csako G., Bielekova B. (2012). Inhibition of LTi cell development by CD25 blockade is associated with decreased intrathecal inflammation in multiple sclerosis. Sci. Transl. Med..

[200] Eken A., Yetkin M.F., Vural A., Okus F.Z., Erdem S., Azizoglu Z.B., Haliloglu Y., Cakir M., Turkoglu E.M., Kilic O. (2019). Fingolimod alters tissue distribution and cytokine production of human and murine innate lymphoid cells. Front. Immunol..

[201] Acar N.P., Tuncer A., Ozkazanc D., Ozbay F.G., Karaosmanoglu B., Goksen S., Sayat G., Taskiran E.Z., Esendagli G., Karabudak R. (2020). An immunological and transcriptomics approach on differential modulation of NK cells in multiple sclerosis patients under interferon-β1 and fingolimod therapy. J. Neuroimmunol..

[202] Thell K., Hellinger R., Sahin E., Michenthaler P., Gold-Binder M., Haider T., Kuttke M., Liutkevičiūtė Z., Göransson U., Gründemann C. (2016). Oral activity of a nature-derived cyclic peptide for the treatment of multiple sclerosis. Proc. Natl. Acad. Sci. USA.

[203] Huang Y., Mao K., Chen X., Sun M.-A., Kawabe T., Li W., Usher N., Zhu J., Urban J.F., Paul W.E. (2018). S1P-dependent interorgan trafficking of group 2 innate lymphoid cells supports host defense. Science.

[204] Hermann R., Krajcsi P., Fluck M., Seithel-Keuth A., Bytyqi A., Galazka A., Munafo A. (2022). Cladribine as a Potential Object of Nucleoside Transporter-Based Drug Interactions. Clin. Pharmacokinet..

[205] Lünemann J.D., Ruck T., Muraro P.A., Bar’Or A., Wiendl H. (2020). Immune reconstitution therapies: Concepts for durable remission in multiple sclerosis. Nat. Rev. Neurol..

[206] Aglas-Leitner F.T., Juillard P., Juillard A., Byrne S.N., Hawke S., Grau G.E., Marsh-Wakefield F. (2022). Mass cytometry reveals cladribine-induced resets among innate lymphoid cells in multiple sclerosis. Sci. Rep..

[207] Lancet T. (2018). End of the road for daclizumab in multiple sclerosis. Lancet.

[208] Croteau D., Tobenkin A., Brinker A., Kortepeter C.M. (2021). Tumefactive multiple sclerosis in association with fingolimod initiation and discontinuation. Mult. Scler. J..

[209] Jeung L., Smits L., Hoogervorst E., van Oosten B., Frequin S. (2020). A tumefactive demyelinating lesion in a person with MS after five years of fingolimod. Mult. Scler. Relat. Disord..

[210] İriş M., Kızılkılıç O., Saip S., Uygunoğlu U. (2024). Recurrent tumefactive demyelination under fingolimod treatment. Neurol. Sci..

[211] Faissner S., Hoepner R., Lukas C., Chan A., Gold R., Ellrichmann G. (2015). Tumefactive multiple sclerosis lesions in two patients after cessation of fingolimod treatment. Ther. Adv. Neurol. Disord..

